# Membrane-Located Expression of Thioesterase From *Acinetobacter baylyi* Enhances Free Fatty Acid Production With Decreased Toxicity in *Synechocystis* sp. PCC6803

**DOI:** 10.3389/fmicb.2018.02842

**Published:** 2018-11-27

**Authors:** Shajia Afrin, Md. Rezaul Islam Khan, Weiyi Zhang, Yushu Wang, Weiwen Zhang, Lin He, Gang Ma

**Affiliations:** ^1^Bio-X-Renji Hospital Research Center, Renji Hospital, School of Medicine, Shanghai Jiao Tong University, Shanghai, China; ^2^Bio-X Institutes, Key Laboratory for the Genetics of Developmental and Neuropsychiatric Disorders (Ministry of Education), Shanghai Jiao Tong University, Shanghai, China; ^3^Shanghai Animal Disease Control Center, Shanghai, China; ^4^Laboratory of Synthetic Microbiology, School of Chemical Engineering and Technology, Tianjin University, Tianjin, China

**Keywords:** membrane scaffold, free fatty acids (FFAs), thioesterase, *Synechocystis* sp. PCC6803, *Acinetobacter baylyi*

## Abstract

It has been previously reported that photosynthetic production of extracellular free fatty acids (FFAs) in cyanobacteria was realized by thioesterases (TesA) mediated hydrolysis of fatty acyl-ACP in cytosol and excretion of the FFA outside of the cell. However, two major issues related to the genetically modified strains need to be addressed before the scale-up commercial application becomes possible: namely, the toxicity of FFAs, and the diversity of carbon lengths of fatty acids that could mimic the fossil fuel. To address those issues, we hypothesized that generating FFAs near membrane could facilitate rapid excretion of the FFA outside of the cell and thus decrease toxicity caused by intracellular FFAs in the cytosolic expression of thioesterase. To realize this, we localized a leaderless thioesterase (AcTesA) from *Acinetobacter baylyi* on the cytosolic side of the inner membrane of *Synechocystis* sp. PCC6803 using a membrane scaffolding system. The engineered strain with AcTesA on its membrane (mAcT) produced extracellular FFAs up to 171.9 ± 13.22 mg⋅L^-1^ compared with 40.24 ± 10.94 and 1.904 ± 0.158 mg⋅L^-1^ in the cytosol-expressed AcTesA (AcT) and wild-type (WT) strains, respectively. Moreover, the mAcT strain generated around 1.5 and 1.9 times less reactive oxygen species than AcT and WT, respectively. Approximately 78% of total FFAs were secreted with an average rate of 1 mg⋅L^-1^⋅h^-1^, which was higher than 0.44 mg⋅L^-1^⋅h^-1^ reported previously. In the case of mAcT strain, 60% of total secreted FFAs was monounsaturated (C18:1) which is the preferable biodiesel component. Therefore, the engineered mAcT strain shows enhanced FFAs production with less toxicity which is highly desirable for biodiesel production.

## Introduction

Given their rapid growth, simple nutrient requirements, ability to incorporate environmental CO_2_ and solar energy, and the recent progress in understanding physiology at the molecular level, cyanobacteria offer a promising host system for fatty acids based biodiesel production as an alternative to fossil and algal fuels (Lardon et al., 2009; Hannon et al., 2010; Liu et al., 2011b; Jin et al., 2014). Biodiesel must adopt the quality of fossil fuel before it can be used in currently available diesel engines. This quality mostly depends on the degree of saturation/unsaturation in the carbon length of fatty acid ester in biodiesel. The ‘cetane value’ is the term widely used to indicate this property of biodiesel. The biodiesel with saturated fatty acid ester, such as ethyl ester of palmitic acid, has a cetane value of 93.1, whereas polyunsaturated fatty acid ester, such as ethyl ester of linolenic acid, has a low cetane value of 39.6 (Knothe et al., 2003). Generally, diesel engines operate well with diesel having a cetane value between 40 to 55. Thus, an ideal biodiesel would be a mixture of both saturated and monounsaturated fatty acid esters with cetane value of more than 40 (Cao et al., 2014). The heterologous expression of thioesterase hydrolyses acyl-ACP to generate free fatty acids (FFAs) and secrete them out of cells in cyanobacteria. For example, thioesterase from *Escherichia coli* (TesA) produces C14:0, C16:0, and C18:0; plant thioesterase, such as Ch FatB2 produces C10:0 and C12:0; Uc FatB1 produces C12:0 and C14:0; and Cc FatB1 generates C14:0 FFAs after expression in *Synechocystis* (Liu et al., 2011b). Despite the success in expressing different thioesterases and the production of FFAs in cyanobacteria, the effort is still needed to produce unsaturated FFAs in an engineered host with minimum toxic effect on it to achieve practical application.

Naturally, cyanobacteria are sensitive to FFAs, especially unsaturated fatty acids (Ruffing and Trahan, 2014). The FFA concentration beyond its solubility limit is known to be toxic and affects cell viability; thus, production cannot be increased after a certain level (Liu et al., 2011b; Ruffing and Trahan, 2014; Kato et al., 2017). For example, *Synechococcus* sp. PCC 7002 is highly susceptible to α-linolenic acid and can withstand only 25 μM in culture media (Ruffing and Trahan, 2014). In addition, the unsaturated fatty acid may produce toxic products, such as hydrogen peroxide or free radical species, by reacting with reactive oxygen species (ROS) and intercalating them in cellular and organellular membrane (Desbois and Smith, 2010). Ruffing found that presence of intracellular FFAs in two FFA-producing engineered strains of *Synechococcus elongatus* PCC 7942, SEO1 and SEO2, produce 11.1% ± 2.3% and 58.1% ± 24% more ROS, respectively, compared with the wild-type (WT) (Ruffing, 2013). Thus, the scattered presence of FFAs in the cytosol of engineered bacteria where thioesterase is expressed may be responsible for ROS generation (Dunlop et al., 2010). Considering this issue, immediate removal of intracellular FFAs after formation in the FFA-producing strain is necessary to alleviate ROS formation.

To improve biofuel production in cyanobacteria by enhancing the removal of intracellular FFAs, a few approaches have been implemented previously to minimize the intracellular FFAs and thus increase extracellular fatty acid production. Liu et al. (2011b) weakened the outer peptidoglycan layer and found that extracellular fatty acid secretion was increased in the SD232 (without peptidoglycan) strain (90.5 ± 6.4 mg⋅L^-1^) compared with SD225 (with peptidoglycan, 83.6 ± 11.4 mg⋅L^-1^). However, this modification made the cell fragile and prolonged the lag phase. In another effort, a synthetic feedback control system based on efflux pump, which can export FFAs outside, has been reported in *E. coli* (Dunlop et al., 2010). However, this system is membrane associated and needs an intricate balance of expression because disproportional expression can affect membrane protein localization and membrane fluidity. Another report showed that fortification of FFAs from the culture using a two-phase system with isopropyl myristate not only enhanced total FFA production (0.64 g⋅L^-1^) but also decreased the intracellular FFAs and corresponding toxicity (Kato et al., 2017). Despite all these achievements, the cost-effectiveness of this system remains undetermined. These studies clarify that removal of toxicity during FFA production can be beneficial to increase production.

The application of artificial scaffolds in cell factory can be advantageous in localizing biochemical reactions in particular organelles to decrease the transit time of the final product, which further benefits the reaction equilibria and kinetics (Horn and Sticht, 2015). Previously, the scaffold system is reported to enhance production of different industrially important products, such as mevalonate (Martin et al., 2003), glucaric acid (Moon et al., 2009), H_2_ (Agapakis et al., 2010), butyrate (Baek et al., 2013), and resveratrol (Conrado et al., 2012). The rational design of scaffold system with modular building blocks is likely to favor the near-membrane FFAs generation so that it could be secreted outside promptly.

In this study, we managed to express a truncated thioesterase (‘AcTesA) from *Acinetobacter* in *Synechocystis* (AcT strain), which excreted a considerable amount of saturated and unsaturated FFAs. The membrane localization of thioesterase is assumed to hydrolyse acyl-ACPs near cell membrane and release FFAs to culture media, which could alleviate toxicity caused by the intracellular accumulation of FFAs. To verify this hypothesis, we fused the truncated AcTesA with the C terminus of Lgt (phosphatidylglycerol: prolipoprotein diacylglycerol transferase), a membrane scaffold protein of *Synechocystis*, and found that the engineered strain not only excreted more fatty acids but also experienced less ROS level. Given the aforementioned reports on cyanobacterial FFA-based biofuel production, we envision that the synthetic membrane scaffold developed in this study can be extended to achieve a robust cyanobacterial host for FFA production.

## Materials and Methods

### Media and Growth Conditions of Bacterial Strain

All strains developed in this study were derived from *Synechocystis* sp. PCC6803 (hereafter described as WT) provided by Prof. Weiwen Zhang (Tianjin University, China). The strains were grown at 30°C in BG-11 medium (both solid and liquid) (Stanier et al., 1971) under continuous illumination of 25 μmol photons m^-2^s^-1^ light, with shaking at 130 rpm for normal growth in a photo-incubator shaker. The mutant culture was supplemented with antibiotics of different concentrations. In the solid culture for plating and transformant selection, 50 μg⋅mL^-1^ kanamycin was added to 1.5% agar plates, which were incubated under continuous illumination of 25 μmol photons m^-2^s^-1^ light at 30°C. The liquid culture was supplemented with 100 μg⋅mL^-1^ kanamycin. All strains were maintained in BG-11 medium with 25% glycerol and stored at -80°C.

### Growth Pattern and Cell Viability Test

Bacterial cell growth in liquid culture was monitored spectrophotometrically or by flow cytometry. The optical density of the cultures was measured at 730 nm using a spectrophotometer (Agilent CARY-60). A total of 1 mL of culture with approximately 1 × 10^5^ cells was stained with 30 nM SYTOX^®^ Green nucleic acid stain (Invitrogen Molecular Probes, Inc.) (Roth et al., 1997) for 20 min at room temperature to detect damaged cells using the BD FACS (fluorescence-activated cell sorting) Aria II (BD Bioscience) flow cytometer with 488 nm excitation and emission collected in a 530/30 bandpass filter or equivalent. The fluorescent cells were sorted and counted as damaged.

### Construction, Transformation and Mutant Generation

Details of the strains and plasmids used in this study are described in Supplementary Table S1. The vectors were constructed by combining normal restriction–digestion–ligation system and the fusion system, following the method described by Zhang et al. (2015). The pBluescript II KS (+) cloning plasmid was used to construct all the vectors used in this study. FFA-producing strains were transformed with pAcT and pmAcT vectors to obtain the mutant AcT and mAcT, respectively. The primers for constructions and genotype verifications are listed in Supplementary Table S2. The details information of the nucleic acid sequence of genes, promoters and others fragments used in this study were listed in Supplementary Table S3. The constructed vectors were transformed in *Synechocystis* using the method described by Liu et al. (2011b). For transformation, *Synechocystis* fresh culture was taken at OD_730_ 0.4–0.6. The collected cells after centrifugation at room temperature were diluted with fresh BG-11 media at around OD_730_ 2.5 with a total volume of 500 μL containing corresponding vector DNA with a concentration of 5–20 μg. The cells were incubated overnight at 30°C under continuous white light illumination at 25 μmol photons m^-2^s^-1^. Then, approximately 200 μL of cells were spread on the BG-11 solid media containing 50 μg⋅mL^-1^ kanamycin and incubated for 7–10 days. Colony appeared on the plate after 4–6 days of incubation. Single colonies were collected and streaked on the BG-11 solid media containing 50 μg⋅mL^-1^ kanamycin for at least five generations to obtain complete segregation. The confirmation of positive mutant was performed by PCR (Liu et al., 2011b), and the positive clone was used to set the liquid culture in 250 mL flask with an antibiotic. Further sequencing was conducted to confirm the mutant strain using the PCR amplified DNA obtained in colony PCR using the primers specific for the inserted gene segments or the deleted region.

### Quantitative Real-Time RT-PCR

The AcT and mAcT strain set to grow in normal condition and then AcT strain induced by adding 1 mM IPTG and placing both of the cultures under normal light conditions for 24 h. RNA was extracted from 15 ml of cell culture using Trizol reagents (Invitrogen, Carlsbad, CA, United States) following the manufacturer’s instruction. One microgram of total RNA was used as starting material for the cDNA generation using GoScript^TM^ Reverse Transcription System (Promega, United States) following the described instructions.

The qRT-PCR was performed applying Lightcycler480 Real-Time PCR system in a 20 μL reaction system containing 10 μL Light cycler^®^480 SYBER^®^Green I master (Roche) mix, 9 μL of template cDNA (100X diluted) and 0.5 μL of each PCR primer (5 picomoL concentration). Three biological along with three technical replications were performed for each sample. Data were analyzed using light cycler software. The data were normalized by using an internal controller, *rnpB* expression pattern. Fold change was calculated comparing between mutants. The significant change had been evaluated by student’s *t-test* applying the software GraphPad PRISM Version 5.01. The primers used for qRT-PCR analysis are also listed in Supplementary Table S2.

### Isolation of Pure Plasma Membrane (PM) and Thylakoid Membrane (TM) Proteins

In AcT strain ‘AcTesA expressed freely in cytosol whereas in mAcT strain fused with an inner membrane protein. We isolated both cytosolic and membranes proteins. Membrane proteins were isolated by following the method described by Norling et al. (1998) and Haigh et al. (2013) (described in Supplementary Method S3) with slight modification. Cells were harvested after induction of 24 h when the OD_730_ reached at around 0.45 (3–5 days of growth) by centrifugation for 10 min at 6700 rpm and 4°C. Total membrane proteins were prepared by suspending collected cells in a 20 mM potassium phosphate (pH 7.8) to a final volume of 5 mL. Acid-washed glass beads from Sigma (diameter 0.425–0.6 μm) were added to the sample tube and shaken in a vortex mixer three times at the highest speed for 2 min with 1 min intervals on the ice and then centrifuged for 1 min at 4000 × *g* at 4°C. The upper cell suspension was collected and centrifuged again at 4000 × *g* and 4 °C for 10 min. Then, the collected supernatant was centrifuged for 30 min at 103000 × *g* at 4°C. The dark blue supernatant was discarded. The pellet of total membrane proteins was rinsed with a buffer containing 0.25 M sucrose and 5 mM potassium phosphate (pH 7.8) and mixed with the same buffer up to 3 mL.

Aqueous polymer two-phase partitioning was applied to separate PM and TM proteins from total membrane proteins. The two-phase systems were prepared from stock solutions of 20% (w/w) Dextran T-500 (Solarbio) and 40% (w/w) polyethylene glycol 3350 (Sigma-Aldrich). Total membranes were applied to a polymer mixture yielding a two-phase system of 5.8% (w/w) Dextran T-500, 5.8% (w/w) polyethylene glycol 3350, 0.25 M sucrose and 5 mM potassium phosphate (pH 7.8). A repartitioning system with the same concentrations (but without membrane sample) was also prepared. Another repartitioning system with 6.2% of both polymers in the same buffer and sucrose medium was prepared.

The partition steps were performed by gently inverting the tubes 35 times at 4°C. Phase settling was done by centrifugation at 1000 × *g* for 4 min at 4°C, and the upper and lower phases were collected separately. The lower and upper phases were repartitioned with upper and lower phases from the (5.8%) repartitioning system, yielding the second upper and lower phase fractions. Another partitioning cycle was conducted to produce third fractions of upper and lower phase. The third lower phase fraction was repartitioned two more times with the 5.8% upper phase to obtain the fifth fraction of the lower phase. The third upper phase fraction was added to a 5.8% lower phase and supplemented with the Dextran (20%) and polyethylene glycol (40%) stock solution to obtain 6.2% of each portioning polymer. After partitioning, the resultant upper phase was repartitioned two more times with the lower phase to obtain the sixth upper phase. At this point, the fifth lower phase and sixth upper phase were diluted with 0.25 M sucrose and 5 mM potassium phosphate (pH 7.8) up to 12 mL and centrifuged for 1 h at 125000 × *g* and 4°C. The pelleted protein dissolved with 0.25 M sucrose and 5 mM potassium phosphate buffer (pH 7.8) containing 1 mM PMSF. The fifth lower phase contained the TM proteins, and the sixth upper phase contained the PM proteins. For cytosolic protein, small volume (15 ml) of cell culture of at log phase was taken to perform a protein assay. Cell harvested by centrifuge at 8000 rpm at RT for 8 min. The supernatant was discarded and the cell pellet dissolved in 1ml BG-11media. 200 μL of the dissolved cell was centrifuged and re-dissolved in 45 μL distilled deionized water into an eppendorf tube and boiled with 5 μl of protein loading buffer for 10 min and kept on ice for 10min. After boiling the sample was centrifuged at 13,000 rpm for 5 min at 4°C. Collected supernatant contained crude protein sample. Protein concentrations of the cytosolic and membrane protein fractions were measured using a Nanodrop spectrophotometer (Nanodrop 1000, Thermo Scientific) set at 280 nm. Proteins were separated by SDS-PAGE using 12% polyacrylamide gel. Equal volumes of protein and protein marker loaded into the wells of the gel, to track the loading and to compare the band size. The gel blotted onto PVDF membranes pre-soaked with methanol. The membrane was blocked with 5% non-fat milk for 1 h and then incubated with the anti-His (EarthOx) primary antibody (Mouse, 1:1000 in 5% non-fat milk) for overnight at 4°C. After washing with TBST (a mixture of Tris-buffered saline and Tween 20), the membrane was incubated with the anti-mouse (Cell Signal Technology) secondary antibody (1:2500 in 5% non-fat milk) for 2 h at room temperature. After washing, the membrane soaked in 1:1 HRP substrate and HRP peroxide for 1 min and then observed the band under chemiluminescence. The target band of only ‘AcTesA is approximately 20 kDa and Lgt-‘AcTesA fused protein is ∼50 kDa.

### FFA Extraction and Measurement

Free fatty acid analysis conducted following the method described by Liu et al. (2011b) with some modification. Hexane used to separate secreted extracellular FFAs from the culture medium, in which intact cells were unable to release FFAs and other lipids. In total, 20 mL of culture was taken at the late-log phase with approximately 10^9^ cells⋅mL^-1^. The culture was acidified by 0.4 mL H_3_PO_4_ (1M) containing 0.4 g of NaCl. The acidification step was briefly maintained to avoid cell disruption. A total of 10 mL of hexane was added to the acidified culture and shaken. After centrifugation for 10 min at 8000 rpm, the upper hexane layer was collected and dried overnight at 40°C. The cell pellet was allowed to dry at -80°C in a freeze dryer. The dried samples from the hexane-dissolved FFA were then completely re-dissolved with methanol (2–3 mL). An equal volume of BF3-methanol was added for methylation of fatty acids. The weight of the freeze-dried cell pellet was measured and recorded as dry cell weight (DCW), which was further used for normalization of FFA production.

The DCW obtained above was also used to extract and analyze intracellular un-secreted FFAs. The dried cell pellet was grinded finely with a mortar and pestle, dissolved in 3 mL chloroform: methanol (2:1) and vortexed for 30 min. The pellet was then centrifuged for 10 min at 8000 rpm, and the supernatant was collected and dried at 40°C. After drying, a stream of nitrogen flow was given to the dried sample to dry out any remaining methanol or chloroform. The subsequent steps were similar to extracellular fatty acid extraction and methylation. The samples were analyzed by GC-MS to determine the extracellular and intracellular FFA amount and profile using WT as the control. The individual FFA were confirmed and compared with retention time and peak areas of standard library and internal standard, respectively (Supplementary Figure S3). The FFAs profile of WT membrane lipids was obtained from Wada and Murata (1990).

Before subjecting to GC-MS analysis, the methylated samples were supplemented with the internal standard tridecanoic acid (C13:0). GC-MS operating conditions were as follows: split ratio 1:20; inject volume 1 μL; helium carrier gas with constant flow rate 30 mL⋅min^-1^; H_2_ 40 mL⋅min^-1^, air 400 mL⋅min^-1^, make-up gas (helium) 5 mL⋅min^-1^ and injector and detector temperature 250°C; and oven temperature started at 150°C, increased at a rate of 10°C min^-1^ to 220°C and maintained for 10 min. Each FFA compound was identified by comparing its retention time with that of the standard. The concentrations in the samples were quantified based on the area under the chromatogram peak in comparison with the standards.

### Cellular ROS Measurement

Cells were grown in standard growth condition for 168 h, and samples were obtained to estimate the total ROS content. The total cellular ROS was measured by using membrane-permeant fluorescence indicator 5-(and-6)-chloromethyl-2′,7′-dichlorodihydrofluorescein diacetate, CM-H_2_DCFDA (Invitrogen, Life Technology), following the method described by Hakkila et al. (2014) and the protocol provided by Life Technology. A total of 1 mL of culture was collected, and CM-H_2_DCFDA was added to the culture with a final concentration of 25 mM. Another 1 mL of culture without the addition of CM-H_2_DCFDA was taken as the control (auto-fluorescence). Both the sample (treated with CM-H_2_DCFDA) and control (without CM-H_2_DCFDA treatment) were incubated for 90 min in complete darkness at 32°C. Then, the cells were washed twice with BG-11 media and re-suspended to a final volume of 0.5 mL with BG-11. A total of 200 μL of cell suspension was pipetted to a white 96-well microtiter plate. Fluorescence from CM-H_2_DCFDA-treated and untreated cells (auto-fluorescence) was measured by using the Synergy 2 Multi-Mode Reader (BioTeK). The excitation and detection emission had been set as 485/20 and 535/20 nm, respectively, with a sensitivity of 50, optic position in the bottom and normal read speed. BioTek’s Gen5^TM^ 1.10 Reader Control and Data Analysis Software was used to set and analyze the data. The ROS content was calculated by employing the following equation: ROS content = (FRT-IRT)-(FRUT-IRUT), where IRT is the initial reading of treated sample, FRT is the final reading of treated sample, IRUT is the initial reading of untreated sample, and FRUT is the final reading of untreated sample.

## Results

### Generation of Mutant Strains and Expression of Recombinant ‘AcTesA Protein

**FIGURE 1:**
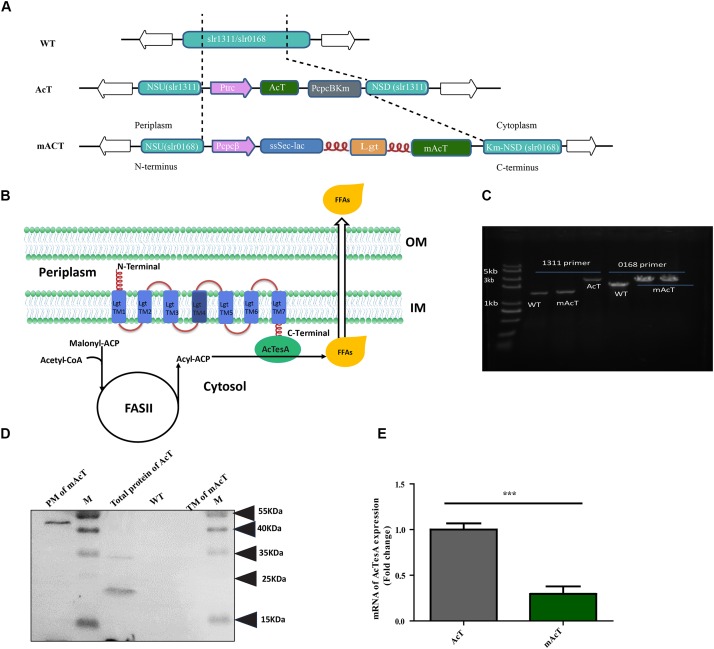
Generation of fatty acid-producing mutant strains using synthetic protein scaffold on the membrane of *Synechocystis* sp. PCC6803. **(A)** Design of gene arrangement in WT, AcT and mAcT strain. The dashed line indicates the neutral sites for the insertion of target genes. In AcT, ‘AcTesA is the truncated thioesterase from *Acinetobacter baylyi*; Ptrc is the promoter; NSU and NSD are the upstream and downstream flanking regions of the *slr1311*, respectively; Km is the kanamycin resistance cassette. In mAcT, PcpcB is the promoter; NSU and NSD are the upstream and downstream flanking regions of the deleted gene, *slr0168*, respectively. In the –ssSec-lac-lgt-‘AcTesA- cassette, ssSec is the signaling sequence; lac is β-lactamase; lgt is phosphatidylglycerol: prolipoprotein diacylglycerol transferase, an inner membrane protein; flexible linker FL3 (red color and coil-shaped) is introduced between proteins to fuse β-lactamase to the N-terminus and ‘AcTesA to the C-terminus of Lgt. **(B)** Hypothetical presentation of the mechanism of membrane-localized ‘AcTesA in generating and facilitating the excretion of FFAs outside the cell. **(C)** PCR confirmation of the insertion in the AcT and mAcT mutant strains. Lane M is a marker. Lanes 1, 2, and 3 are bands of DNA amplified using primers NSUF (1311) and NSDR (1311) to confirm the –AcT-PcpcKm- insertion. In lanes 4–6, the primers NSUF (0168) and NSDR (0168) were used to amplify the –PcpcBssSecLacLgtAcTesAKm-cassette insertion to confirm the insertion with around 3.7 kb in size. WT is the band without target insertion around 1.9 and 1 kb with the primer pair NSUF/NSDR (0168) and NSUF/NSDR (1311), respectively. **(D)** Western blot analysis of fractionated protein from mAcT and total crude AcT. Here, M is the marker; PM, plasma membrane; TM, thylakoid membrane; WT, total crude protein from WT *Synechocystis*. MW of ‘AcTesA is approximately 20 kDa and Lgt-‘AcTesA fusion is approximately 50 kDa. **(E)** qRT-PCR analysis of expression of ‘AcTesA. Data shown here are the mean ± SE from biological triplicates with 4 technical replicates and “^∗^” represents statistical significance as indicated by Student’s *t*-test with a maximum *p*-value of < 0.05.

*Acinetobacter* naturally accumulates wax derived from FFAs (Reiser and Somerville, 1997). Many species of *Acinetobacter* have been found with the efficient intracellular FFA-producing ability (Reiser and Somerville, 1997; Ukey et al., 2017). Hydrolysis of acyl-ACP by thioesterase is the most common reaction to produce intracellular FFAs. *Acinetobacter baylyi* was reported to have four different thioesterases, namely, TesA, B, C and D (hereafter AcTesA, AcTesB, AcTesC, and AcTesD to make different from other thioesterase; Ac represent *Acinetobacter*). Amongst them, only AcTesA and AcTesC exhibit thioesterase activity. A truncated AcTesA was expressed in *E. coli* and was used to produce long chain (C16:0 to C18:0), unsaturated FFAs (C18:1) and short chain FFAs (Ukey et al., 2017). This AcTesA from *Acinetobacter baylyi* composed of 212 amino acids (AA) in length, which includes a 30 AA-long N-terminus leader sequence that helps the native protein to localize in the periplasmic space of cells (Zheng et al., 2012). Interestingly, AcTesA was found to have high sequence similarity [37.97% AA identity and similar catalytic triad formation (Ser10-Asp154-His157)] with *E. coli* thioesterase, TesA (Zheng et al., 2012). Despite this similarity, they have fairly different substrate specificities, with AcTesA hydrolysing the acyl-ACP of saturated and unsaturated acyl group having a carbon length of C8 to C18 (Zheng et al., 2012; Ukey et al., 2017) and TesA preferring to hydrolyse saturated acyl-ACP. To realize high production efficiency of fatty acids from carbon dioxide, we expressed ‘AcTesA (denotes the leaderless AcTesA) in *Synechocystis* sp. PCC6803. We constructed two *Synechocystis* strains named AcT and mAcT. For the AcT strain, we inserted the 546-bp ‘AcTesA with the N-terminus 6XHis tag along with the kanamycin resistance cassette (KanR) into the neutral site (*slr1311*) (Lagarde et al., 2000; Kuchmina et al., 2012; Bentley et al., 2014; Tsujimoto et al., 2018) of the *Synechocystis* genome under IPTG inducible promoter, Ptrc (Figure 1A). This strain was developed to express ‘AcTesA in the cytosol to facilitate random hydrolysis of intracellular acyl-ACP. For the mAcT strain, the ‘AcTesA gene containing 6XHis at the N-terminus was fused to the C-terminus of the Lgt protein (Pailler et al., 2012), which was expressed under light-responsive promoter (PcpcB) by inserting into the neutral site (*slr0168*) of the *Synechocystis* genome. PcpcB is chosen because it is relatively less stronger promoter than Ptrc (Markley et al., 2015; Wang et al., 2018) and has the ability to express for a longer period under the normal light condition, thus saving the cell from a sudden excessive load of ‘AcTesA on the membrane. The gene *lgt* encodes phosphatidylglycerol: prolipoprotein diacylglycerol transferase (Lgt), which catalyzes one of the three steps of the lipoprotein biosynthetic pathway. To understand the functional similarity of *Synechocystis* Lgt with others, we performed multiple alignments with Lgt from *E. coli* and other gram-negative bacteria, whose structure and function have recently been illustrated in details (Pailler et al., 2012; Mao et al., 2016) (Supplementary Figure S1). The cyanobacterial Lgt protein is 283 AA-long and has 23.2% identity and 42% positivity in alignment with Lgt from *E. coli*, which is a protein of 291 AAs in length. The recent crystallographic investigation of *E. coli* Lgt revealed that Lgt has seven transmembranes (TM1–TM7) domains. Similar to *E. coli*, the TM4 of Lgt from *Synechocystis* has the signature motif [LVI]^(-3)^ [ASTVI]^(-2)^ [GAS]^(-1)^ C^(+1)^ for lipoprotein binding (also called lipid box). The TMs span across the inner membrane in a way that the N-terminal faces to the periplasmic space and the C-terminal to the cytoplasm. Thus, fusing ‘AcTesA with the C-terminal of Lgt protein is supposed to localize the thioesterase near the membrane at the cytoplasmic side (Figure 1B).

The WT *Synechocystis* was transformed with constructed vector pAcT and pmAcT to generate AcT and mAcT strain, respectively. The transformed AcT and mAcT strains were selected on BG-11 plate supplemented with kanamycin (50 μg⋅mL^-1^). Several single colonies were taken and cultured by streaking on the BG-11 plate supplemented with 50–200 μg⋅mL^-1^ kanamycin. The integration of the ‘AcTesA and Lgt-‘AcTesA gene in the WT *Synechocystis* genome and complete segregation were confirmed by PCR using genomic DNA as a template (Figure 1C). To assess the expression of ‘AcTesA in the AcT strain, the initial OD_730_ of the culture was set to ∼0.01, and the culture was allowed to grow in normal light with 25 μmol photons m^-2^⋅s^-1^, shaking at 130 rpm under 30°C until the OD_730_ reached 0.40–0.45 (which takes around 30 h). Subsequently, 1 mM of freshly prepared IPTG was added and the culture was then placed in normal growth condition for more than 24 h. After collecting the cells, we extracted total protein and performed Western blot analysis using anti-His antibody. The results showed a clear band of ‘AcTesA at around 20 kDa (Figure 1D), which confirming the expression of ‘AcTesA in AcT strain.

In the mAcT strain, ‘AcTesA is expected to attach to Lgt and bind with the cell membrane. We grew the cells in the normal growth condition mentioned above and continue the growth until the OD_730_ reached 0.45–0.50. After cell collection and sonication, we fractionated the cells and separated the thylakoid membrane (TM), plasma membrane (PM) and cytosolic fraction (details are provided in the Section “Materials and Methods”). Western blot analysis using anti-His antibody marked the protein at around 50 kDa, which is the approximate size of the fused ‘AcTesA and Lgt in the PM fraction (Figure 1D). Generally, the leaderless AcTesA (‘AcTesA) should be solubilized and localized in the cytosol (Zheng et al., 2012). To confirm this in our case, we isolated total membrane from AcT (after IPTG induction), mAcT and WT strain and performed western blot using anti-His antibody. We observed that only the membrane from mAcT strain retains the ∼50 kDa protein which is ‘AcTesA-Lgt fusion protein size(Supplementary Figure S2). To quantify ‘AcTesA expression in both AcT and mAcT strain, we performed qRT-PCR of cDNA made from isolated mRNA and found that the expression of ‘AcTesA in AcT strain is higher than the mAcT strain (Figure 1E).

### FFA Production and Secretion: mAcT Produces High Amounts of Extracellular Fatty Acids

**FIGURE 2:**
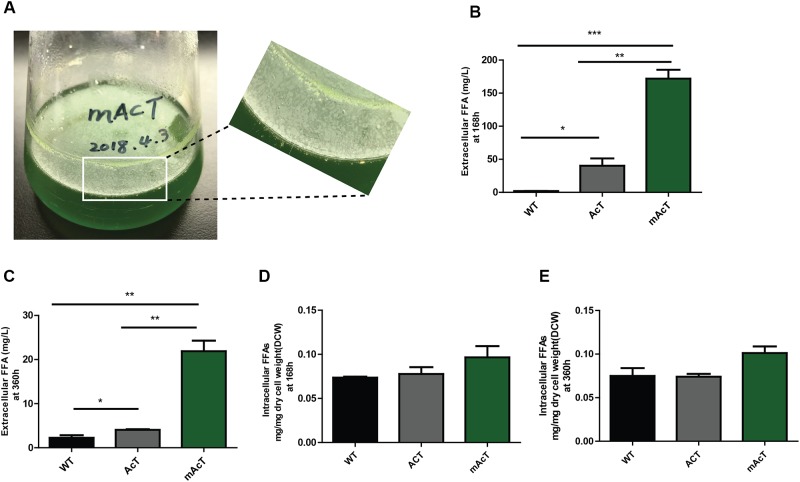
Production and secretion of FFAs. Cultures were grown at 30°C in a BG-11 medium under continuous illumination of 25 μmol photons m^-2^ s^-1^ light. **(A)** Droplets of FFAs appeared on the culture medium of mAcT after 168 h. GC-MS-based analysis of **(B)** extracellular FFAs (FFAs) at 168 h, **(C)** extracellular FFAs (FFAs) at 360 h, **(D)** intracellular FFAs at 168h and **(E)** intracellular FFAs at 360 h from the culture of WT, AcT and mAcT set in a 250 mL flask. Data shown in **(B–E)** are the mean ± SE from biological triplicates, and ‘^∗^’ represents statistical significance as indicated by Student’s *t*-test with a maximum *p*-value of <0.05.

The extracellular FFA production was monitored after induction of normal-grown cell by adding 1 mM IPTG in AcT culture and continue the growth of the both AcT and mAcT culture under the normal growth condition for different time intervals (168 and 360 h). A few oil droplets, possibly FFAs (Figure 2A); appeared after 120 h on the surface of the culture of mAcT strain. The similar oil droplets were reported in another engineered *Synechocystis* strain harboring exogenous thioesterase from *E. coli* (Liu et al., 2011b). Figures 2B,C showed that the total FFAs secreted outside of the cells in the AcT and mAcT strains were approximately 10 and 40 times higher than the WT strain at 168 h, respectively. The mAcT strain secreted 171.9 ± 13.22 mg⋅L^-1^ FFAs in the flask culture media compared with AcT and WT, which were found to secrete 40.24 ± 10.94 and 1.904 ± 0.158 mg⋅L^-1^ FFAs, respectively, at 168 h (Figure 2B). However, when the same culture continued to grow for 360 h and extracellular FFAs were analyzed, the result showed a similar trend of change between WT, AcT and mAcT. The results also showed that 78% of total FFAs were extracted from the culture media. The result implies that AcT strain produce less extracellular FFAs than mAcT strain (Figures 2B,C) despite the fact that ‘AcTesA expression is higher in AcT strain (Figure 1E). Following the analysis by Kato et al. (2016), we calculated the average rate of FFA secretion as 1 mg⋅L^-1^⋅h^-1^, which was approximately two times higher than another engineered *Synechocystis* sp. PCC 6803, SD277 (Liu et al., 2011b). By comparing the recent efforts (Supplementary Table S4) of biodiesel production in engineered cyanobacteria, we found our strategy is simple and significantly increased the secretion of FFAs with less cellular toxicity.

To determine if the expression of ‘AcTesA in the cytosol (AcT strain) and on the membrane (mAcT strain) could affect the intracellular fatty acid, we extracted and analyzed the intracellular fatty acids. The results showed no significant difference in terms of fatty acid amount among the WT, AcT and mAcT strains at 168 h. In longer cell culture, at around 360 h the intracellular FFAs amount changed as a similar pattern that observed at 168 h with no significant difference among the strains (Figures 2D,E). The GC-MS result of extracellular and intracellular FFAs are summarized in Table 1, 2.

**Table 1:** The secreted extracellular and intracellular FFAs calculation.

Strains	Time	Cell density	Extracellular FFAs	Extracellular FFAs	Intracellular FFAs	Intracellular FFAs	Total FFAs
	(h)	(Cells/mL)	(mg/L)	(mg/cell)	(mg/cell)	(mg/mg DCW)	(mg/L)
**WT**	168	1.6137 × 10^8^	1.90 ± 0.15	1.18 × 10^-11^	10.75 × 10^-11^	0.07 ± 0.053	19.26 ± 1.38
	360	2.4212 × 10^8^	2.25 ± 0.58	0.92 × 10^-11^	27.08 × 10^-11^	0.07 ± 0.0451	67.84 ± 10.94
**AcT**	168	1.9553 × 10^8^	40.24 ± 10.94	2.05 × 10^-10^	18.46 × 10^-11^	0.07 ± 0.384	76.35 ± 9.37
	360	2.5393 × 10^8^	4.05 ± 0.15	1.59 × 10^-11^	35.67 × 10^-11^	0.07 ± 0.163	94.64 ± 22.42
**mAcT**	168	2.0342 × 10^8^	171.96 ± 13.22	8.45 × 10^-10^	22.60 × 10^-11^	0.09 ± 0.637	217.94 ± 20.75
	360	2.6735 × 10^8^	21.88 ± 2.39	8.18 × 10^-11^	43.39 × 10^-11^	0.10 ± 0.387	137 ± 9.6

*N.B., the data calculated from 3 biological replications. Here DCW represent Dry-cell-weight, FFA, Free fatty acids*.

**Table 2:** The ratio between intracellular FFA and dry cell weight calculated from cells grown under normal growth condition.

				Fold Change		
Time	WT	AcT	mAcT	mAcT/WT	ACT/WT	mAcT/ACT
	(mg/mg DCW)	(mg/mg DCW)	(mg/mg DCW)			
168 h	0.07	0.08	0.09	1.31	1.05	1.24
360 h	0.07	0.07	0.10	1.35	0.98	1.36

### mAcT Produced High Percentage of Unsaturated Fatty Acids

**FIGURE 3:**
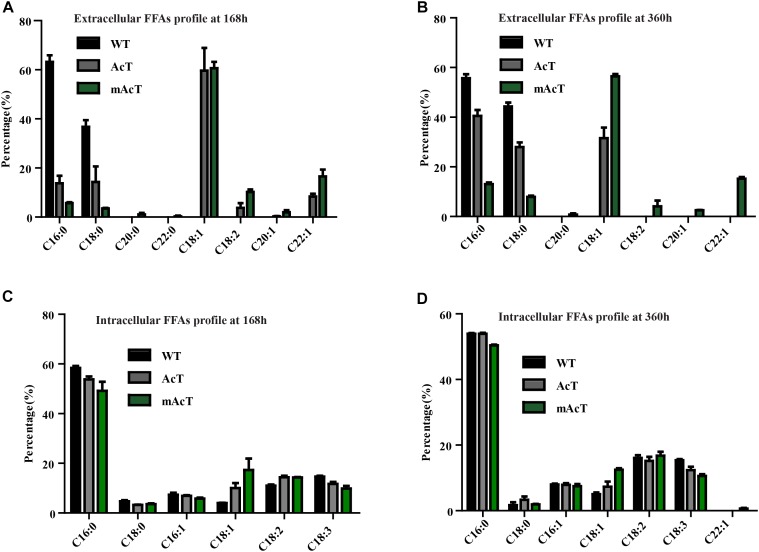
Fatty acid profile of FFA-producing strain. Cultures were grown at 30°C in a BG-11 medium under continuous illumination of 25 μmol photons m^-2^ s^-1^ light. FFA compositions were analyzed by GC-MS. **(A)** Extracellular FFA profile at 168 h, **(B)** extracellular FFA profile at 360 h, **(C)** intracellular FFA profile at 168 h and **(D)** intracellular FFA profile at 360 h. Data shown here are the mean ± SE from biological triplicates.

The fatty acid pool analysis from AcT and mAcT Synechocystis showed that extracellular FFAs included at least nine different fatty acids with various carbon lengths (16:0, 16:1, 18:0, 18:1, 18:2, 20:0, 20:1, and 22:1) (Figures 3A,B). When we compare among WT, AcT and mAcT strains, the extracellular FFA distribution pattern was saturated, C16:0 and C18:0, in the WT, whereas both mutants showed a large proportion of unsaturated fatty acids C18:1, C18:2, and C22:1 (Table 3). Eighty percent of the total FFAs were dominated by the 16 and 18 carbon FFAs in AcT and mAcT at 168 h. However, the intracellular FFA profile was not different amongst WT, AcT and mAcT strains (Figures 3C,D).

**Table 3:** FFAs profile of WT, AcT, and mAcT strains analyzed by GCMS.

Percentage of FFAs (%)							
	
FFAs	WT			AcT		mAcT	
	Total	Extracellular	Intracellular	Extracellular	Intracellular	Extracellular	Intracellular
	Lipid	FFAs	FFAs	FFAs	FFAs	FFAs	FFAs
C16:0	52	63.7	58.4	15.2	53.2	5.7	48.7
C18:0	t	36.3	4.6	17.4	3.3	3.5	3.6
C20:0	Nd	Nd	Nd	Nd	Nd	1.2	Nd
C22:0	Nd	Nd	Nd	Nd	Nd	0.3	Nd
C16:1	3	Nd	7.4	Nd	6.8	Nd	5.8
C18:1	5	Nd	4	54.7	10.8	60.6	18.0
C18:2	11	Nd	11	4.5	14.5	10.2	14.3
C18:3	29	Nd	14.6	Nd	11.4	Nd	9.6
C20:1	Nd	Nd	Nd	0.4	Nd	1.9	Nd
C22:1	Nd	Nd	Nd	7.8	Nd	16.6	Nd
Total	100	100	100	100	100	100	100

*Here t, Trace amount; nd, not detected. The fatty acid percentages in WT membrane lipids were taken from Wada and Murata*.

Moreover, 80.5% of the total FFAs in the mutant strain (mAcT) were unsaturated fatty acids, which was higher than that reported by Cao et al. (2010, 2014) in *E. coli*. To get a balanced cetane value, a mixture of saturated and monounsaturated fatty acids are highly demanded (Islam et al., 2013; Cao et al., 2014). In mAcT strain, we found a higher amount of monounsaturated fatty acids (MUFAs), more than 60% of the total extracellular secreted fatty acid was C18:1, suggesting that AcTesA could be favorable in the production of monounsaturated FFAs in Synechocystis.

### Optimization of Extracellular FFA Production

**FIGURE 4:**
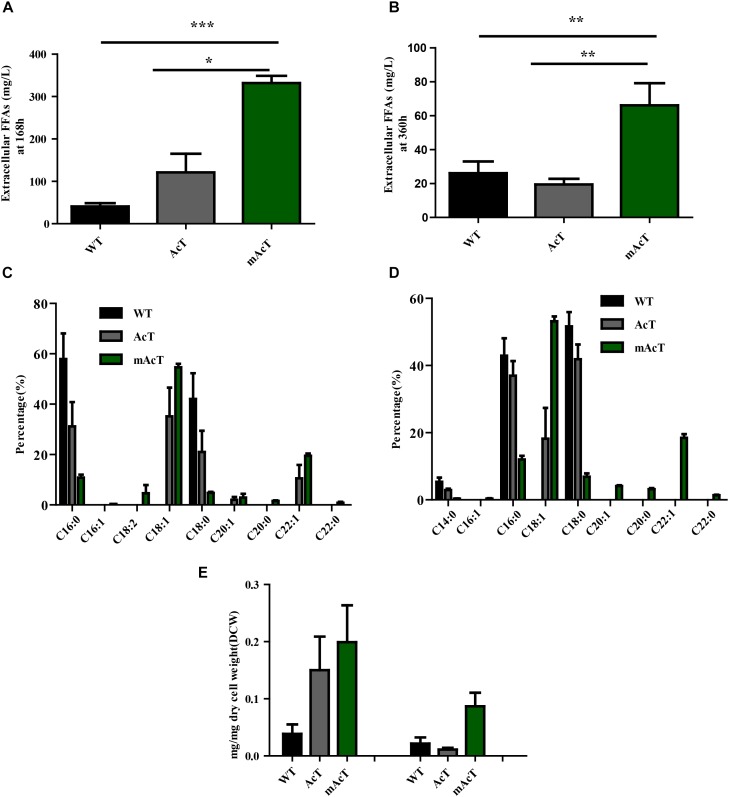
Effects of CO_2_ on extracellular FFA production. Cultures were grown at 30°C in a BG-11 medium under continuous illumination of 50 μmol photons m^-2^ s^-1^ light and bubbled with 1% CO_2_-enriched air. Measurement of **(A)** extracellular FFAs at 168 h and **(B)** extracellular FFAs at 360 h. Profiles of **(C)** extracellular FFAs at 168 h and **(D)** extracellular FFAs at 360 h of WT, AcT and mAcT. **(E)** Extracellular FFA amount after normalization with dry cell weight (DCW) at two time points, 168 and 360 h. Data shown in **(A–E)** are the mean ± SE from biological triplicates, and ‘^∗^’ represents statistical significance as indicated by Student’s *t*-test with a maximum *p*-value of < 0.05.

To understand the effect of CO_2_ on the extracellular FFA production of the AcT and mAcT strain, we set the culture at 30°C in a BG-11 medium under continuous illumination of 50 μmol photons m^-2^⋅s^-1^ light and bubbled with 1% CO_2_-enriched air and continued the cell growth for 400 h. The sample collection and analysis of extracellular FFAs were conducted using the method described above (FFA production and secretion). The results showed that the mAcT strain secreted 331 ± 9.576 mg⋅L^-1^ FFAs into the flask culture media compared with AcT (121 ± 9.260 mg⋅L^-1^) and WT (40 ± 0.752 mg⋅L^-1^), which are approximately three and eight times higher, respectively, at 168 h (Figure 4A). The concentration of extracellular FFAs was two times higher than that obtained in normal growth condition in the case of AcT and mAcT strains but was considerably higher too in the WT. However, when the same culture was continued for up to 360 h, the results of extracellular FFA analysis showed a similar trend of change between WT and mAcT, whereas AcT showed significantly lower concentration (Figure 4B). These results implied that the addition of CO_2_ in the culture media might play a vital role in the secretion of intracellular FFAs by rapidly growing cells with increased biomass. To confirm this finding, we incubated AcT and mAcT in the presence of 1% continuous CO_2_ flow and then extracted and analyzed extracellular fatty acids. We found that the total extracellular FFAs in the mAcT were around five and two times higher than those in the WT and AcT at 168 h, respectively. The results also demonstrated the same pattern of change when the growth continued to 360 h (Figure 4E). We compared these results with those obtained in normal growth condition and found that although the addition of CO_2_ substantially increased the biomass, the ratio between FFA and dry cell weight was not considerably changed between the two culture systems with or without the addition of CO_2_ (Table 4). The fatty acid pool analysis indicated that the extracellular FFAs had similar patterns of fatty acid distribution as those found in normal growth condition (16:0, 16:1, 18:0, 18:1, 18:2, 20:0, 20:1, and 22:1) at two time points: 168 and 360 h (Figures 4C,D).

**Table 4:** The ratio between extracellular FFA and dry cell weight calculated from cells grown under normal and CO_2_ enriched conditions.

					Fold Change		
Time	Growth condition	WT	AcT	mAcT	mAcT/WT	ACT/WT	mAcT/ACT
		(mg/mg DCW)	(mg/mg DCW)	(mg/mg DCW)			
168 h	Normal	0.008	0.09	0.36	43.60	11.51	3.78
	In CO_2_	0.038	0.15	0.19	5.14	3.88	1.32
360 h	Normal	0.002	0.003	0.02	7.83	1.38	5.67
	In CO_2_	0.02	0.01	0.08	4.03	0.51	7.78

### mAcT Strain Produced Less ROS and Improved Cell Growth

**FIGURE 5:**
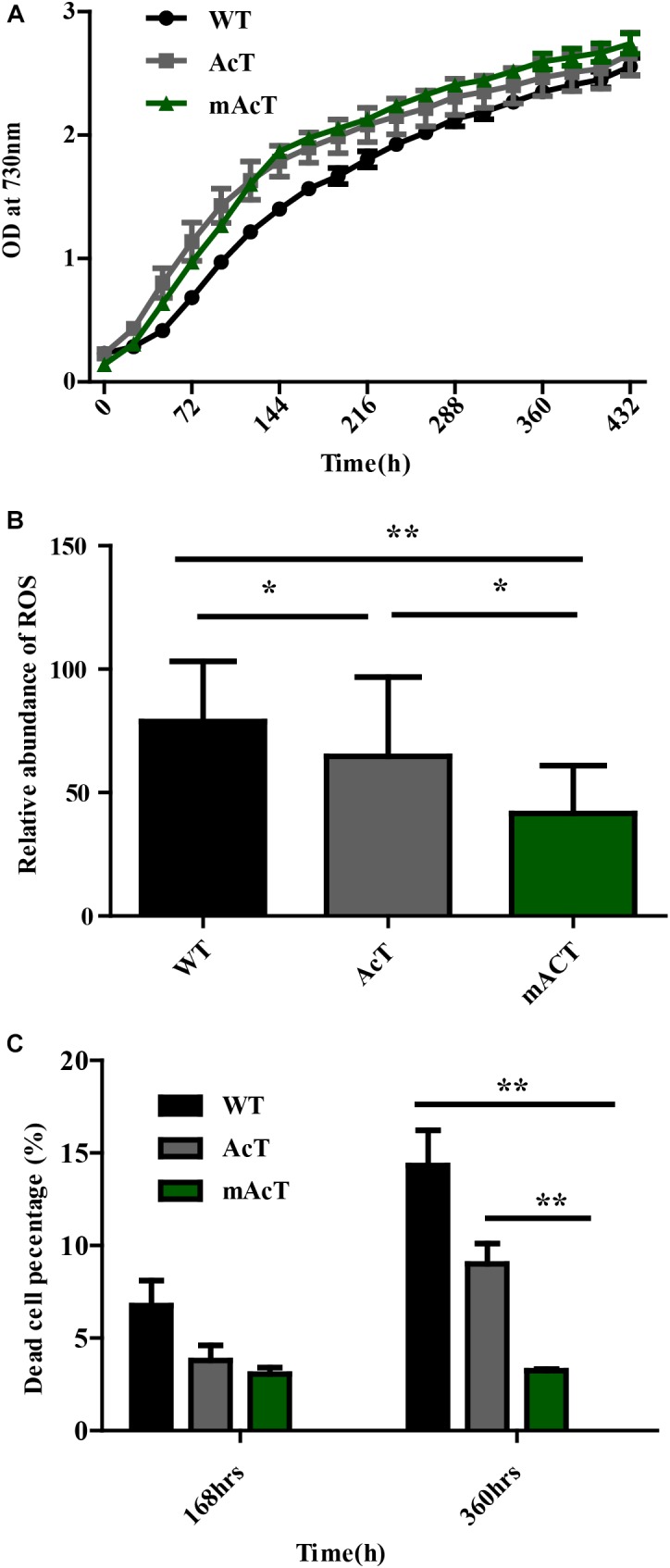
Effects of FFA production on cell survivability. **(A)** Growth pattern of fatty acid-producing strains. The culture was grown in 250 mL culture flask at 30°C in a BG-11 medium under continuous illumination of 25 μmol photons m^-2^s^-1^ light. OD was measured at 730 nm. Data shown here are the mean ± SE from biological triplicates. **(B)** Cellular toxicity and ROS generation in WT, AcT and mACT strains at 168 h. Data shown here are the mean ± SE from five biological replications along with four technical replications and ‘^∗^’ represents statistical significance as indicated by Student’s *t*-test with a maximum p-value of < 0.05. **(C)** Percentage of damaged cells of WT, AcT and mAcT strains sorted by flow cytometry using SYTOX green nucleic acid stain. Data shown here are the mean ± SE from biological triplicates.

A previous study demonstrated that FFA accumulation is toxic to cells (Desbois and Smith, 2010; Kato et al., 2016). To assess this finding, we first determined the growth curve by measuring OD value at 730 nm. We observed that the growth of AcT and mAcT strains was better than that of the WT after induction at the second day. Notably, the mAcT strain showed a higher growth rate than the AcT strain after induction (Figure 5A). To investigate more on the toxicity, we measured total ROS using membrane-permeated fluorescence indicator 5-(and-6)-chloromethyl-2′,7′-dichlorodihydrofluorescein diacetate, CM-H_2_DCFDA, following the protocols described by Khan et al. (2016) (Figure 5B). We observed that the mAcT strain generated less ROS than WT and AcT at 240 h. The relative abundance of ROS in the mAcT strain was 41.6, whereas WT and AcT showed 78.85 and 64.7, respectively. However, at 360 h, the total ROS content in the mAcT strain was two and three times lower than those in the AcT and WT strains, respectively. Thus, the result suggests that mAcT strain will improve growth and is able to decrease ROS generation which is considered as one of the obstacles in FFAs producing strain.

We then determine whether a low ROS content in mAcT was beneficial to cell survival. We measured the dead cell percentage of WT, AcT and mAcT strains by FACS (Figure 5C) using SYTOX^®^ Green dye which can only penetrate the dead cells to differentiate dead cells from live cells (Supplementary Figure S4). We found that the mAcT culture comprised a lower percentage of dead cells (only 3%) than the WT and AcT strains at 168 h. Notably, a longer culture duration increased the difference in dead cell percentage between mAcT (3.2%), AcT (9%) and WT (14.3%); mAcT culture comprised five and three times lower dead cell percentage than WT and AcT culture, respectively. The results indicated that the mAcT strain survived longer than other strains.

**FIGURE 6:**
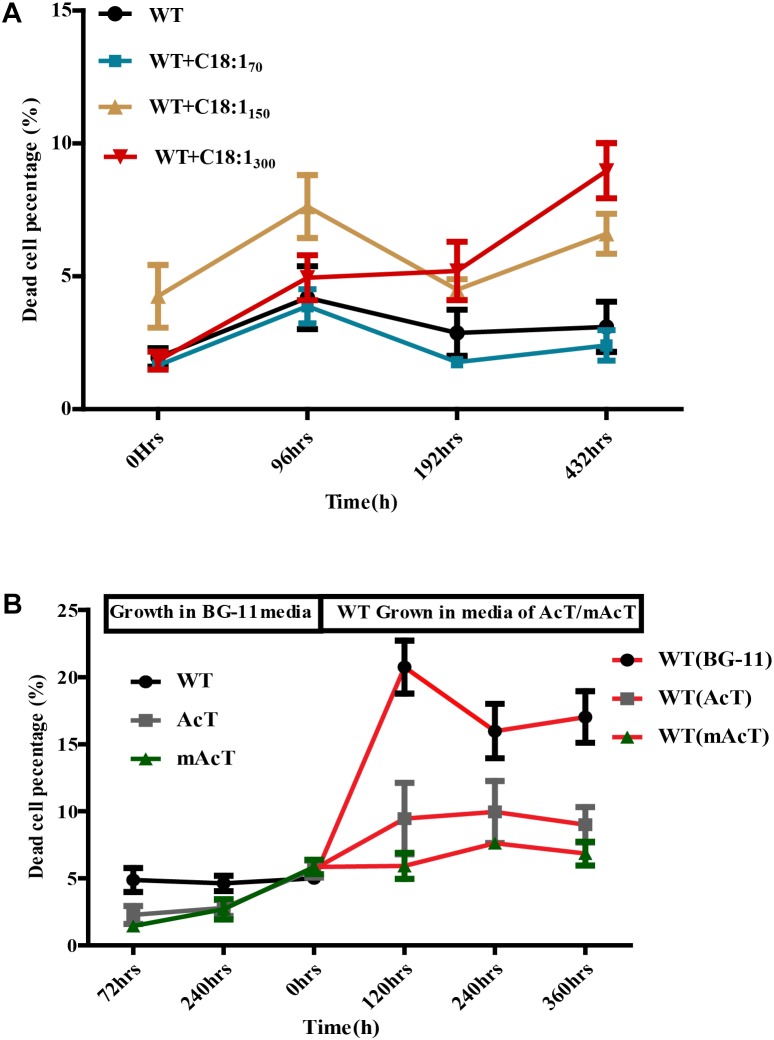
Effect of exogenously added C18:1 FFA and extracted media of mutant strain on WT cells. **(A)** Effects of exogenously added C18:1 fatty acid on the cell survivability of FFA-producing strain. Cells were grown either with or without C18:1 unsaturated fatty acid with different concentrations (70 mg⋅L^-1^ to 300 mg⋅L^-1^) in medium, and the damaged cell percentage was determined by FACS at different time points. **(B)** Role of membrane scaffold on cell survivability. The WT cells were grown in the absence (WT without MS media) or presence (WT in mAcT/AcT media) of media from mAcT/AcT strains (MS media) after 10 days of normal growth in BG-11 media. Data shown here are the mean ± SE from biological triplicates.

To understand the underlying causes of longer survival of mAcT strain, we first assumed that the secreted MUFA could play an important role here. To investigate this, we added different concentrations (70 mg⋅L^-1^ to 300 mg⋅L^-1^) of oleic acid (18:1) into WT culture at 48 h (OD_730_ 0.4) and found no remarkable change in dead cell percentage in long-term incubation despite the initial effect where it showed increased dead cell percentage in the first 96 h (Figure 6A). This result led us to hypothesize that it may not be only single MUFA but a combination of FFAs secreted by mAcT strain could be involved in longer survival of the cell. To test it, we cultivated AcT and mAcT strain for 10 days, extracted the culture media and then inoculated fresh WT cell. The dead cell percentage was counted at different time points, it was found that WT in mAcT-extracted-media showed lower dead percentage in all the time points in comparison with the WT cells grown in AcT-extracted-media and BG-11 media (Figure 6B). The result together suggested that not only the MUFAs but also other factors could influence the longer survivability of mAcT strain.

To understand better the photosynthetic capacity of the WT, AcT and mAcT strain, we tested Chlorophyll a (Chl a) content (Supplementary Method S1) and observed that the mAcT strain does not differ with the WT and AcT strain (Supplementary Figure S5A). We further tested if the membrane localization of AcTesA in mAcT strain affect the ATP synthase ability of cells given that *Synechocystis* consist ATPase on its membrane. A combination of osmotic shock and detergent treatment applied to the collected cells to discharge cellular ATP and reacted with luciferase (details in Supplementary Method S2). A standard curve was prepared using different concentrations of ATP against the intensity of luminescence it can produce in presence of luciferase and luciferin (Supplementary Figure S5B). The slope of the linearly increasing luminescence against the concentration of ATP was used to quantify cellular ATP. We found that mAcT strain does not differ with the AcT and WT strain in terms of cellular ATP synthetic activity (Supplementary Figure S5C). Extracellular ATP was also measured to understand if the AcTesA anchoring on membrane caused damage of the membrane. Hypothetically, if the membrane is damaged, the ATP may come out of the cell. Thus, the presence of ATP outside of the cell can be a good indicator of membrane damage. We found that mAcT culture media consist slightly lower ATP than the AcT strain (Supplementary Figure S5D) and WT but they are not statistically significant (*p*-value higher than 0.05 in student’s *t*-test) indicating that AcTesA expression has not affected the membrane integration in mAcT strain.

## Discussion

The fatty acid synthesis in the FASII pathway resulted in the formation of fatty acyl-ACP with different carbon lengths of the acyl group. These acyl-ACPs can be hydrolysed by the heterologous expression of thioesterase and the release of FFA into the cytoplasm. The FFA can cross the cell membrane into the culture media by two ways: either by specific transporters (Kato et al., 2015) or by passive diffusion against a concentration gradient (Abumrad et al., 1998). The diffusion of FFA across the membrane can be limited by different factors, such as the diffusion coefficient of membrane (Ruffing, 2014); culture temperature, membrane fluidity and protein load on the membrane (Sarcina et al., 2003); and outer layer, including peptidoglycan layer in cyanobacteria (Smarda et al., 2002). Liu et al. (2011b) suggested that deletion of the outer peptidoglycan layer can diffuse FFAs out of the cell by 60% (SDS232 90.5 ± 6.4 mg/L to SDS 246 146 ± 21 mg/L) more than the cell with intact peptidoglycan (Liu et al., 2011b). Based on these previous findings, we hypothesized that generating FFAs near the membrane would generate a potential concentration gradient favoring diffusion of FFAs out of the cells. Moreover, removal of fatty acyl-ACP from the reaction can shift the chemical equilibrium, according to the Le Châtlier principle, to accelerate the accumulation of fatty acids.

To verify this hypothesis, we anchored AcTesA on the inner membrane (mAcT strain) (Figures 1A,B) and found enhanced FFA secretion ability compared with its cytosolic expression strain (AcT) (Figures 2B,C). Amongst all the previous studies on FFA-producing cyanobacterial cells, the dAS1T strain of *Synechococcus elongatus* PCC 7942 and SD277 strain of *Synechocystis* sp. PCC 6803 are highly productive; they secrete FFA concentrations of approximately 0.64 g/L and 200 mg/L in the external medium and have an average secretion rate of 1.5 and 0.44 mg L^-1^ h^-1^, respectively (Liu et al., 2011b; Kato et al., 2017). The mAcT strains constructed in this study showed a relatively higher rate of FFAs excretion (1 mg L^-1^ h^-1^), which is two times higher than SD277 but lesser than the dAS1T strain, with 171.9 mg/L final concentration of secreted FFAs under normal growth condition.

Thus, the result suggested that enhanced FFAs production in mAcT is because of the higher rate of FFAs secretion due to the localisation of ‘AcTesA on the membrane. We found that both AcT and mAcT mutant strains had increased amounts of FFAs in media but unchanged intracellular FFAs. Thus, the results suggested that the secretion of FFAs in the mAcT strain changes the intracellular reaction equilibrium, which favors the production of more fatty acids by possibly reducing the feedback inhibition of acyl-ACP.

Notably, AcTesA expression in *Synechocystis* resulted in different FFA acid pools than TesA expression in other cyanobacteria and TesA or AcTesA expression in *E. coli*; around 60% of the secreted FFA were monounsaturated (Figures 3A,B) (Liu et al., 2011a,b; Zheng et al., 2012; Wijffels et al., 2013). Thus, AcTesA shows different substrate specificity in *E. coli* and *Synechocystis*. This fact is in line with the previous report that thioesterase from different sources can generate FFAs with different carbon lengths in another host. For example, UcfatB1 of *Umbellularia californica* hydrolyses 12 carbon acyl-ACP in the native host but the heterologous expression in *E. coli* would target unsaturated acyl-ACP (Voelker and Davies, 1994). Interestingly, the heterologous expression of UcfatB1 in cyanobacteria hydrolyses only 12 carbon saturated acyl-ACP, suggesting that substrate specificity of thioesterase varies depending on the host (Liu et al., 2011b). How does AcTesA expression targets a large set of unsaturated fatty acids is yet to be investigated. One possible explanation is that the *Synechocystis* membrane requires several unsaturated fatty acids (especially tri-unsaturated) relative to *Synechococcus* sp. PCC 7002 and *Synechococcus elongatus* PCC 7942; thus, availability of unsaturated acyl-ACP as a substrate for AcTesA is more than the saturated acyl-ACP. However, there is no study on the activity of desaturase on acyl-ACP in *Synechocystis*; most of the cyanobacteria desaturase work on the acyl group attached to the membrane lipid. Thus, it would be interesting to study how the expression of AcTesA generates unsaturated FFAs in *Synechocystis* and *E. coli*.

We observed that the accumulation of extracellular FFAs in the media was considerably decreased in long-term (Figures 2B,C). A similar trend was also observed even in the presence of carbon dioxide (Figures 4A,B). We suppose that the FFAs producing mutants are taking back some of secreted FFAs after long incubation when the culture media is exhausted of nutrient. Indeed, both the AcT and mAcT strains still retained the genetic background of membrane-located acyl-acyl carrier synthetase (encoded by *slr1609*) protein, which was previously reported to uptake FFAs from the media and activate subsequent catabolism (von Berlepsch et al., 2012). In most of the previous studies, thioesterase is usually expressed by deleting the slr1609 gene to produce extracellular FFAs. But, deletion of this gene resulted in slower growth and increased cell death (Liu et al., 2011b). In the present study, we aimed to improve FFAs excretion from the cell without perturbing the cell physiology. However, the result suggests that though it is possible to increase FFAs secretion in mAcT strain for a certain period, it is not possible for a long duration in the current genetic background. It would be interesting to see how mAcT strain response in *slr1609* deletion background.

Toxicity is the main problem in fatty acid-based biofuel production in microbial hosts. The defense mechanisms of cells against toxicity vary in different species of cyanobacteria. In most cases, ROS produced under toxicity can generate signals to activate the defense mechanism of cells. Therefore, the cellular ROS level in any given condition is a good indicator of cellular physiological status. Moreover, Ruffing (2013) suggested that fatty acids can produce ROS. Indeed, previous reports suggested that FFAs secretion into the media can cause toxicity to cells by generating ROS (Liu et al., 2011b; Ruffing and Jones, 2012; Ruffing, 2013), and short-chain fatty acids are more toxic than long-chain ones (Royce et al., 2013). Amongst long-chain fatty acids, tri-unsaturated fatty acids were found to be more toxic in cyanobacteria (Ruffing and Trahan, 2014). However, a recent analysis showed that *Synechocystis* could better tolerate the toxic effect of unsaturated fatty acids than other model cyanobacteria. Ruffing and Trahan (2014)revealed that at least 25 μM of α-linolenic acid (18:3) is necessary to inhibit the growth of cyanobacteria (Ruffing and Trahan, 2014), which is much higher than the concentration we obtained (Figures 3C,D). Thus, the probable cause of lower ROS generation in mAcT strain is because of either (i) producing longer chain and unsaturated FFAs, lower than the toxic level, or (ii) rapid excretion of FFAs generated near membrane that helps to minimize ROS generating intracellular FFAs. However, the addition of exogenous unsaturated FFA (18:1) into the culture media of WT failed to show any difference in growth (Figure 6A) excluded the first possibility. No marked difference was observed when WT was grown in the media collected from mAcT-grown culture (Figure 6B). Thus these results led us to suggest that it is probably the rapid excretion of intracellular FFAs in mAcT strain that is favorable to minimize intracellular FFAs level and thus a low level of ROS. This cellular status of lower ROS content of mAcT justified the increased survival of mAcT strain against toxicity.

Together these results indicated that the cellular toxicity in mAcT cell was alleviated by secreting most FFAs in the media. The intracellular FFA accumulation by cleaving cytosolic acyl-ACP can produce FFA in the cytosol, which can cause significant ROS generation. Balancing the rates of FFA production and excretion in mAcT sustained the excretion of FFA for a long period of time. The RND-type export system with the capacity of FFA excretion in *S. elongates* PCC 7942 (Kato et al., 2015) can be useful for improving fatty acid-producing strains. However, the enhancement of passive efflux or the active FFA export was not sufficient to fix the growth defect of the FFA-producing strains (Kato et al., 2015, 2016). The membrane scaffold anchoring and localization of thioesterase near membrane in mAcT strain overcome those limitations. However, it still needs to be investigated that how this localization generates a different pool of fatty acids.

## Conclusion

In this study, we successfully constructed a synthetic protein scaffold on the membrane, carrying the heterologous ‘AcTesA enzyme in genetically engineered *Synechocystis* PCC6803, which can secrete high amounts of FFAs into media from cells. The mutant showed a distinct FFA profile with the highest concentration of MUFAs ever reported in cyanobacteria. In addition, less cellular toxicity was caused by the efficient secretion of FFAs. Moreover, our study presented a new strategy of using the membrane as a scaffold for FFA production, which can be potentially applied to other pathways or products with slight modification.

## Statistical Analysis

The data obtained were analyzed using the software GraphPad Prism 5. All data were expressed as means ± standard error. Student’s *t*-test was used for pairwise comparisons. *P* < 0.05 was considered statistically significant.

## Availability of Data and Materials

All data generated or analyzed during this study are included in this published article with supporting materials (supplementary four tables, five figures and three methods of this work can be found in the online version of the paper).

## Author Contributions

GM conceived and designed the experiments. SA performed the experiments. SA, MK, YW, and GM analyzed the data. SA, MK, WyZ, YW, WwZ, LH, and GM contributed reagents, materials, and analysis tools. SA and GM wrote the paper.

## Conflict of Interest Statement

The authors declare that the research was conducted in the absence of any commercial or financial relationships that could be construed as a potential conflict of interest.
