# Supplementary material for: Membrane-Located Expression of Thioesterase From Acinetobacter baylyi Enhances Free Fatty Acid Production With Decreased Toxicity in Synechocystis sp. PCC6803
Source: Front Microbiol. 2018 Nov 27;9:2842. doi: 10.3389/fmicb.2018.02842 (PMC6277518; doi:10.3389/fmicb.2018.02842)
Supplement: Supplementary file 1 [file Data_Sheet_1.docx]

# Supplementary materials

# Membrane-located expression of thioesterase from *Acinetobacter baylyi* enhances free fatty acid production with decreased toxicity in *Synechocystis* sp. PCC6803

Shajia Afrin ^1, 2^, Md. Rezaul Islam Khan ^2^, Weiyi Zhang^3^, Yushu Wang ^2^, Weiwen Zhang ^4^, Lin He ^2^*, and Gang Ma ^1, 2^*

^1^ Bio-X-Renji Hospital Research Center, Renji Hospital, School of Medicine, Shanghai Jiao Tong University, Shanghai 200240, P.R. China;

^2^ Bio-X Institutes, Key Laboratory for the Genetics of Developmental and Neuropsychiatric Disorders (Ministry of Education), Shanghai Jiao Tong University, Shanghai 200240, P.R. China;

^3^ Shanghai Animal Disease Control Center, Shanghai 201103, P.R. China;

^4^ Laboratory of Synthetic Microbiology, School of Chemical Engineering & Technology, Tianjin University, Tianjin 300072, P.R. China.

Details of the constructed vectors and strains are described in the Table S1. The primers for constructions and genotype verification are listed in the Table S2, along with the primers used for qRT-PCR based detection of ‘AcTesA expression in mutant strains. Details of the gene sequences we used in this study are listed in the Table S3. Some recent efforts to produce biodiesel in cyanobacteria are summarised in the Table S4. We performed multiple sequence alignments of Lgt from *Synechocystis* and other gram-negative bacteria to understand the functional and structural similarity, which showed in the Fig S1. The localisation of ‘AcTesA in AcT strain was confirmed by western blotting which showed in Fig S2. Free fatty acids (FFAs) extracted from mutants were analysed using GC-MS technique. The individual FFA were confirmed and compared with retention time and peak area of standard library and internal standard, respectively. The total ion chromatograms of obtained FFAs were shown in Fig S3. The Figure S4 showed damaged cell percentage at different time points measured by FACS after SYTOX green staining. The Figure S5 described cellular physiological activity in terms of ATP synthesis, membrane damage and Chlorophyll a content. Details of each figure described in the legend following the figure. Methods for measurement of chlorophyll a content, ATP synthesis efficiency test and total membrane protein isolation described in method S1, S2 and S3.

**Table S1.** Strains and plasmids constructed and used in this study.

| **Constructed vectors** | **Corresponding strain and relevant characteristics** |
| --- | --- |
| P^AcT^(pBS-NSU1311-Ptrc-AcTesA-PcpcB-Km-NSD1311) | **AcT**  Inducible FFAs secreting strain; leaderless thioesterase gene ‘AcTesA from *A. baylyi* is controlled by IPTG inducible promoter Ptrc |
| P^mAcT^ (pBS-NSU0168-PcpcB-ssSecLacLgt-AcTesA-PcpcB-Km-NSD0168) | **mAcT**  FFAs secreting strain; *A* leaderless thioesterase gene ‘AcTesA from *A. baylyi* is fused with *Synechocystis sp.* PCC6803 transmembrane protein Lgt, controlled by light inducible promoter PcpcB |

**
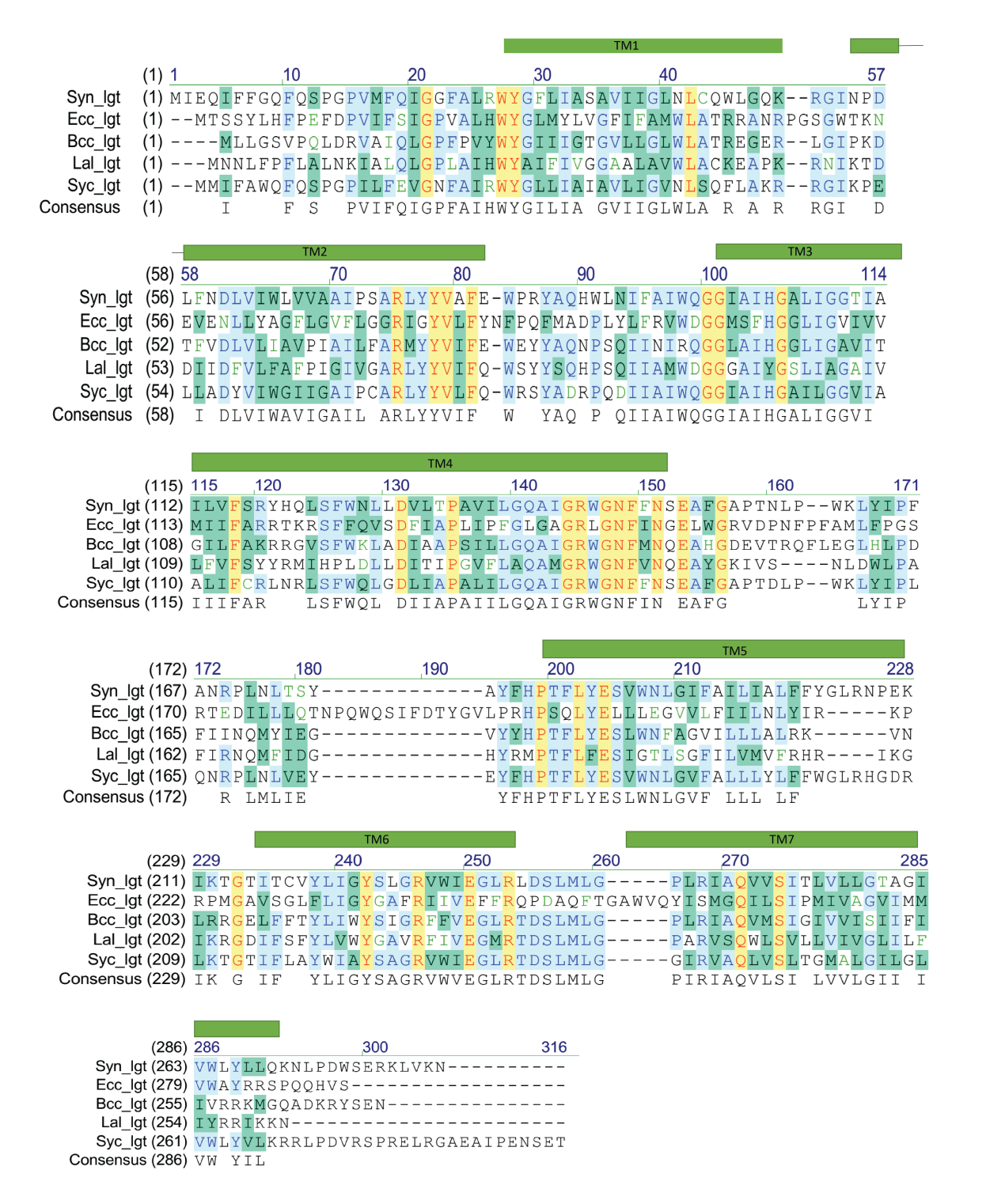
**

**Figure S1**: **Multiple sequence alignment of *Synechocystis* sp. PCC 6803 Lgt with other gram-negative bacteria**. Here Syn= *Synechocystis* sp. PCC 6803, Ecc= *Escherichia coli*, Bcc=*Bacillus cereus*, Lal= *Lactococcus lactis* and *Syc= Synechococcus elongatus* PCC 7942 denoted the comparative bacteria. The cyanobacterial Lgt protein is 283 AA acids long and has 23.2% identity and 42% positivity in alignment with Lgt from *E. coli* that are a protein of 291 AA in length. The crystallographic investigation of *E. coli* Lgt revealed that Lgt has 7 transmembranes (TM1 to TM7) domains. Similar to *E. coli*, the TM4 of Lgt from *Synechocystis* has the signature motif [LVI] ^(-3)^ [ASTVI] ^(-2)^ [GAS] ^(-1)^ C ^(+1)^ for lipoprotein binding lipid box. The yellow backgrounds indicate the identical amino acid residues.

**
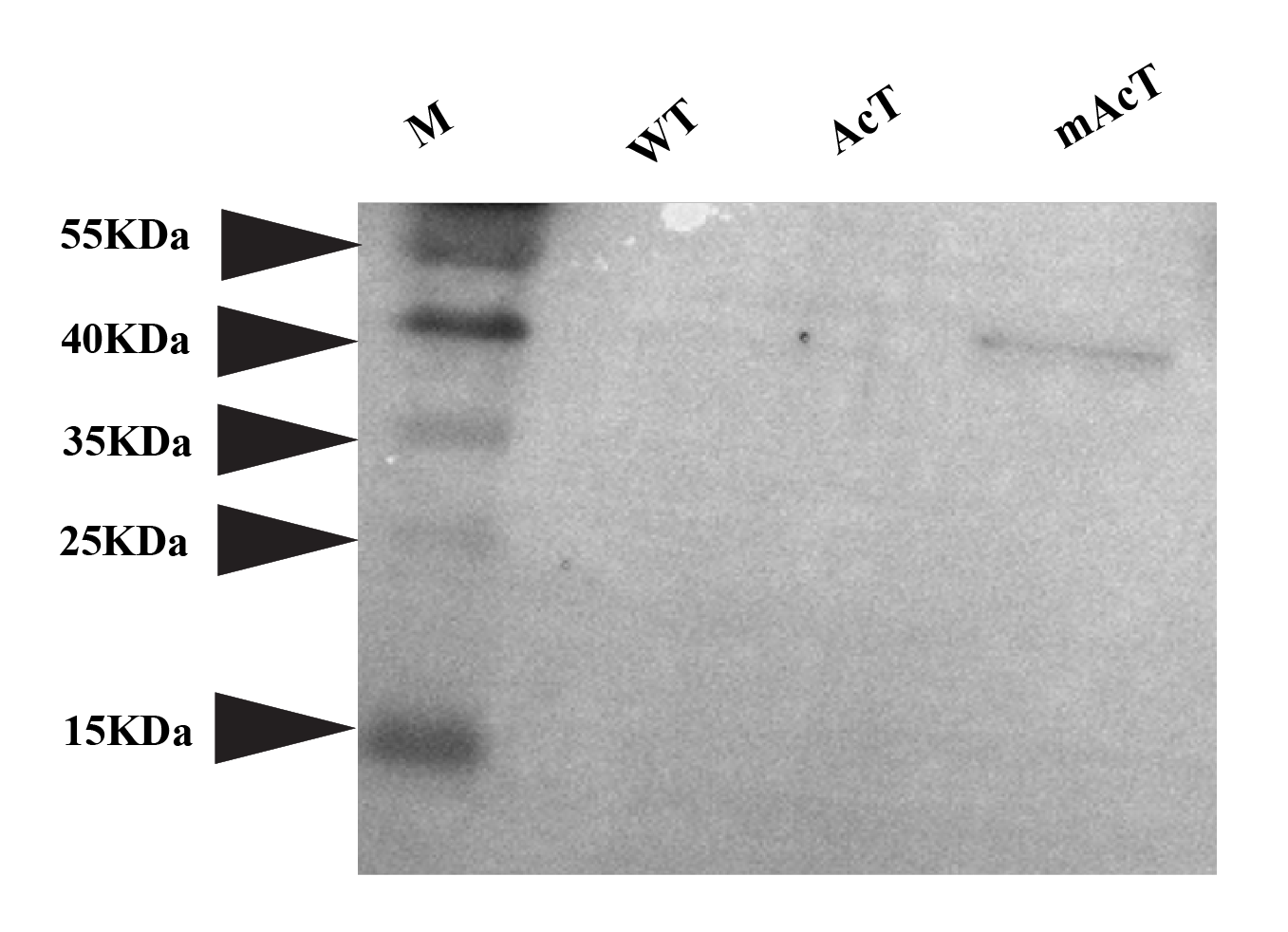
**

**Figure S2.** **Western blot analysis of membrane proteins from mAcT and AcT strains**. M is the marker; WT, membrane protein from WT *Synechocystis* sp. PCC 6803; AcT, mutant strain without membrane scaffold where ‘AcTesA freely expressed in the cytosol as soluble protein; mAcT, with membrane scaffold where ‘AcTesA fused with Lgt membrane protein. MW of ‘AcTesA is approximately 20 kDa and Lgt-‘AcTesA fusion is ~50 kDa.

**
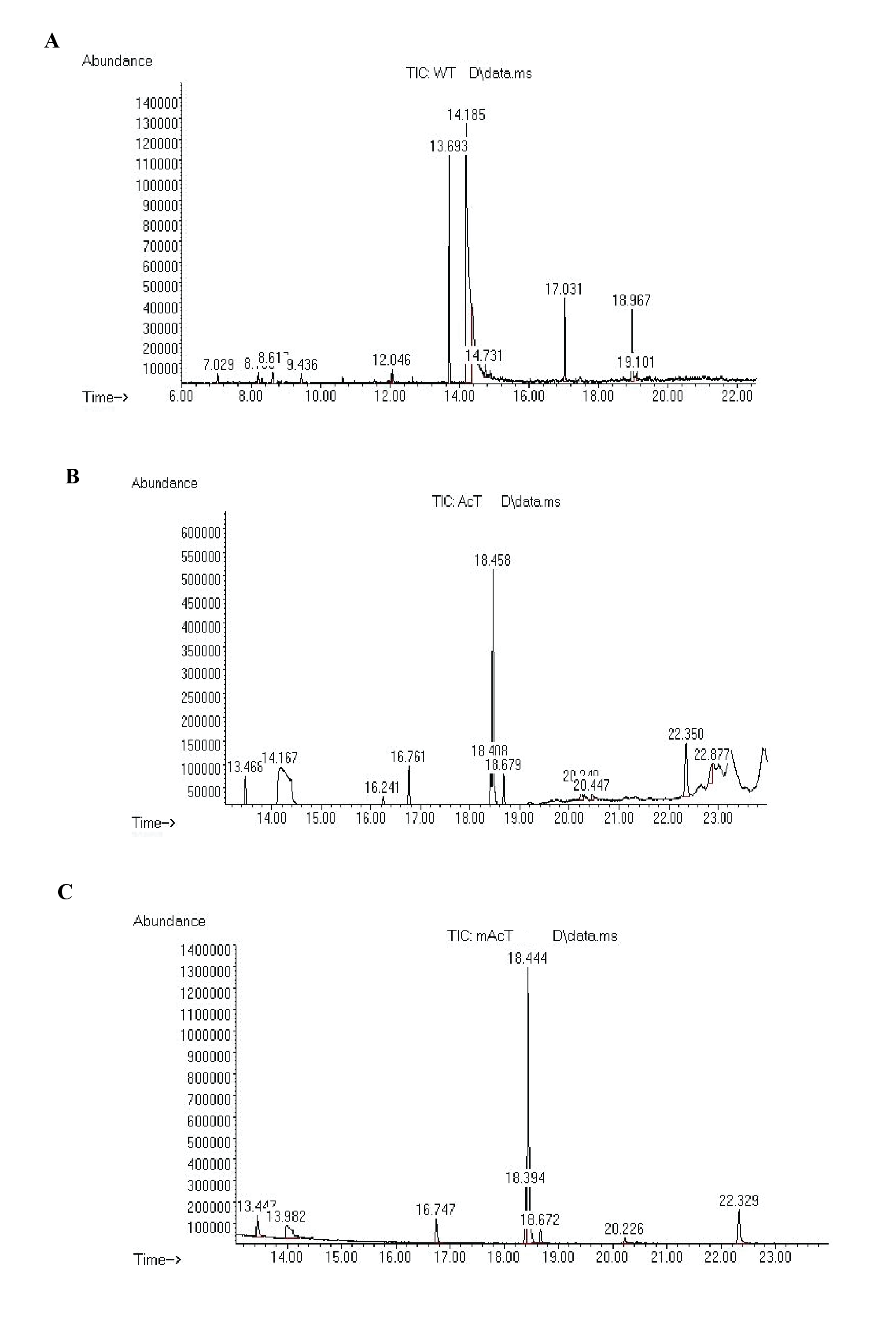
**

**Figure S3. The GCMS analysis of extracellular FFAs**. Total ion chromatogram of extracellular FFAs A) in WT B) in AcT and C) in mAcT strains at 168 hrs. The individual fatty acids were confirmed by comparing their retention times with those obtained from the NIST08 online library. The concentration of each fatty acid was calculated by comparing the peak area of individual FFAs with given internal standard (IS) peak area.

**
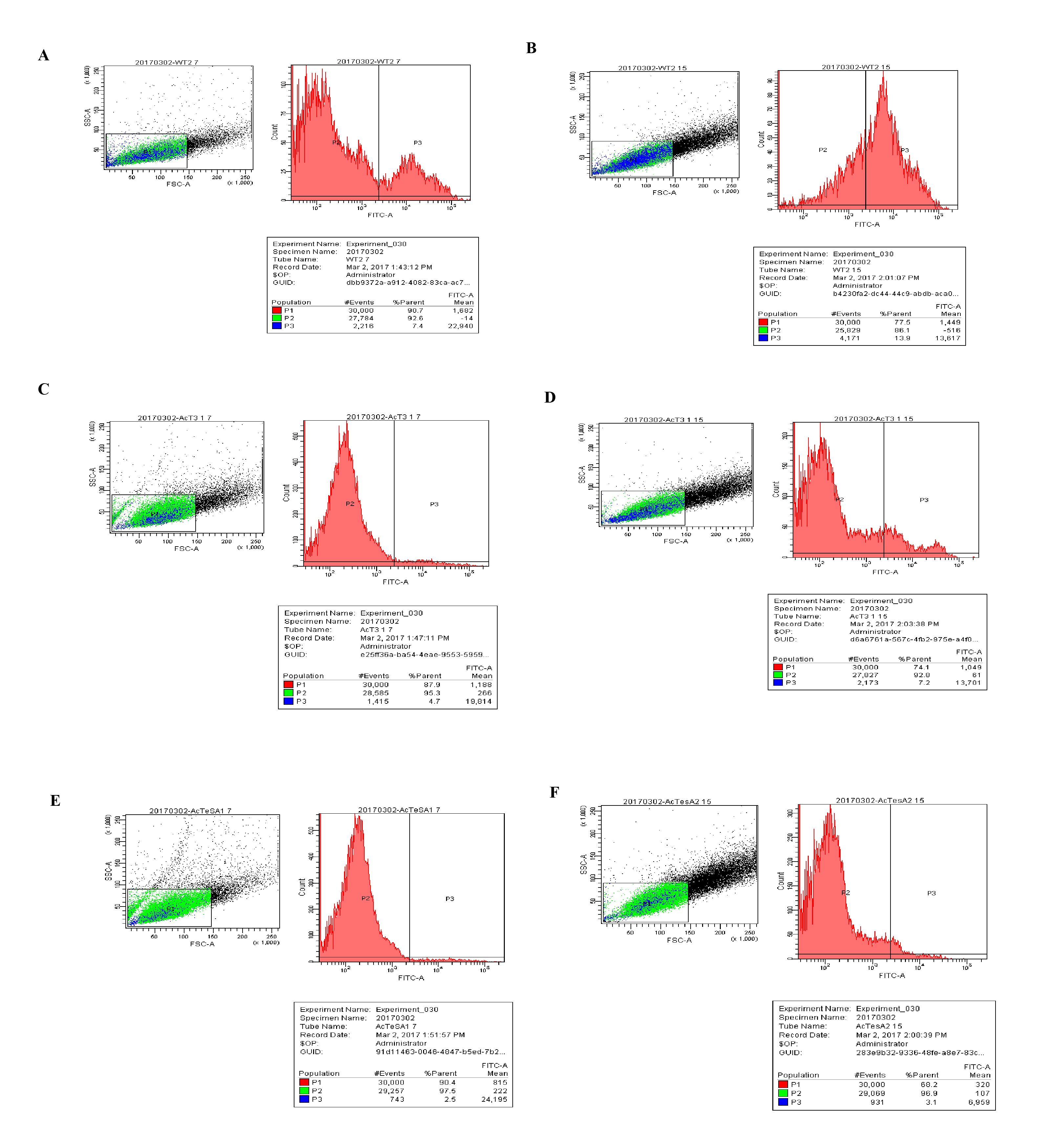
**

**Fig. S4: Damaged cell measurement by flow cytometry**. The cell damage measured during the growth of WT, AcT and mAcT at 30°C in BG-11 medium under continuous illumination of 25 μmol photons m^−2^ s^−1^ light. Cell membrane damaged at different time point 168 h and 360 h were measured by SYTOX green staining following the protocol provided by the company. The percentage of the dead cell at the 168 h culture of A) WT C) AcT and E) mAcT and 360 h culture of B) WT D) AcT and F) mAcT strains. Here, P1, P2 and P3 represent; total cell number, living cells and dead cells respectively.

**
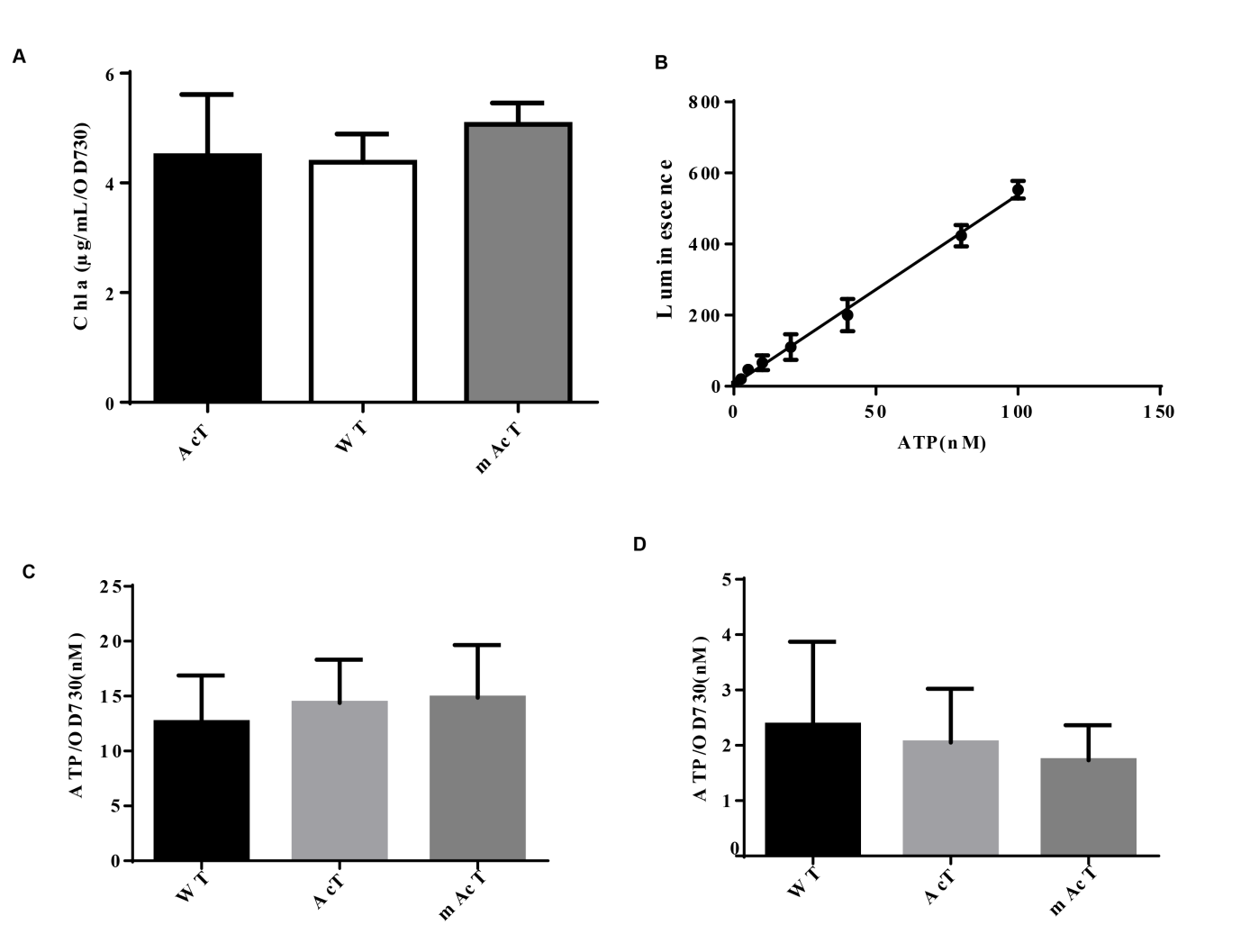
Figure S5.** **Effects of membrane localization of ‘AcTesA on cell physiological activity.** A) Chlorophyll a content B) Luminescence of standard ATP solutions. C) Intercellular and D) Extracellular ATP concentration. Data shown here are the mean ± SEM from 3 biological and 3 technical replications and statistical significance was calculated by Student’s t-test with a maximum p-value of P < 0.05.

**Table S2.** Oligonucleotides used for the construction and genotype verification of the mutants. Sequences are aligned from the 5′ end to the 3′ end.

| **Primer** | **Sequence (5'to3')** |
| --- | --- |
| **Primers used for vectors construction** | |
| SalI-cpcF | ACGCGTCGACGTTATAAAATAAACTTAACAAATCTAT |
| EcoRI-cpcR | CCGGAATTCAATTAATCTCCTACTTGACTTTATG |
| EcoRI-ssSecF | CCGGAATTCTTTAAAGGAGGTTAAGTGTTAAACAAATCTGTTCAGATCC |
| ssSecR | CGGAAATGTTGAATACTCAT AGCCTGGGCGGGGGTTGTA |
| lacF | GCCTCACTGATTAAGCATTGGATGAGTATTCAACATTTCCG |
| BamHI-lacR | CGCGGATCCCCAATGCTTAATCAGTGAGGC |
| BamHI-lgtF | CGCGGATCC**ACTAGAGCTGAGGCCGCCGCAAAAGAAGCAGCAGCTAAGGAAGCTGCGGCGAAG**GCCGAAGGAAGATTAATGATTGAGC |
| XbaI-lgtR | CTAGTCTAGAATTTTTTACCAATTTTCGCTCCG |
| XbaI-GFPF | CTAGTCTAGA**ACTAGAGCTGAGGCCGCCGCAAAAGAAGCAGCAGCTAAGGAAGCTGCGGCGAAG**ATGCGTAAAGGAGAAGAAC |
| NotI-GFPR | AAGGAAAAAAGCGGCCGCTTAGTGGTGATGATGGTGATGTTTGTATAGTTCATCCATGCC |
| KpnI-NSUF(0168) | CGGGGTACCCAACTATGGCTTTGATGG |
| SalI-NSUR(0168) | ACGCGTCGACGTCCCCTTTGAGGATAAT |
| NotI-KmF | AAGGAAAAAAGCGGCCGCGTTATAAAATAAACTTAACAAATCTAT |
| KmR-NSD | GGCGAACTGCGGCTTTTAGAAAAACTCATCGAGCATC |
| Km-NSDF(0168) | GCTCGATGAGTTTTTCTAAAAGCCGCAGTTCGCCAAAT |
| SacI-NSDR(0168) | CGAGCTCCTCCTCCGCCGAGAAAATC |
| AcT-XbaI | GCTCTAGA**ACTAGAGCTGAGGCCGCCGCAAAAGAAGCAGCAGCTAAGGAAGCTGCGGCGAAG**ATGAAAACCATTCTTATCTTAGGCG |
| AcT-NotI | ATAAGAATGCGGCCGC*ATGATGATGATGATGATG*TTATAAAGCGCCTTTAATATATGGG |
| KpnI-1311UF | CGGGGTACCGAATCGCTTCCAATAGTCGTAG |
| XhoI-1311UR | CCCTCGAGATTTTTTGGTCACATTGTCCCT |
| XhoI-AcTF | CCGCTCGAG**GAGCTGTTGACAATTAATCATCCGGCTCGTATAATTTTAAAGAGGAGAAA**ATGAAAACCATTCTTATCTTAGG |
| PstI-AcTR | AAAACTGCAGTTATTACTA*ATGATGATGATGATGATG*TAAAGCGCCTTTAATATATGGG |
| PstI-CpcBF | AAAACTGCAG GTTATAAAATAAACTTAACAAATCTAT |
| CpcBR | TTTTCGTTCCACTGAGCGTAATTAATCTCCTACTTGACTTTATG |
| KanaF | ACGCTCAGTGGAACGAAAA |
| BamHI-KanaR | CGGGATCCGAAAAACTCATCGAGCATC |
| BamHI-1311DF | CGGGATCCCTGGGCTGATGTATTGAACC |
| SpeI-1311DR | CCACTAGTTCCACCAGTTCCAACAAATC |
| **Primers used for confirmation of AcTesA insertion after segregation** | |
| **Primer** | **Sequence (5'to3')** |
| AcT F | AACTGCAGATGAAAACCATTCTTATCTTAGGCG |
| AcT R | CGGGATCCTTATAAAGCGCCTTTAATATATGGG |
| NSUF(1311) | CGGGGTACCGAATCGCTTCCAATAGTCGTAG |
| NSDR(1311) | CCACTAGTTCCACCAGTTCCAACAAATC |
| NSUF(0168) | CGGGGTACCCAACTATGGCTTTGATGG |
| NSDR(0168) | CGAGCTCCTCCTCCGCCGAGAAAATC |
| **Primers used for qRT-PCR** | |
| AcTF1 | 5'CAGGGCTGGGTCGCTTTATT3' |
| AcTR1 | 5'ACCAAGCTCAATGACCACCA3' |
| AcTF2 | 5'CCAGTGTAAGTGGGGAAACCA3' |
| AcTR2 | 5'ATCATTTGAGGCGGTTGTCCT3' |
| AcTF3 | 5'AGCACAGCCAAAAGGCAAAAT3' |
| AcTR3 | 5'CTTTTGTGTCCAGCCACACC3' |
| rnpBF | 5' TCACAAACCACAGCGGCCTA3' |
| rnpBR | 5' GGGCGTTACCCAGCAAGTTT3' |

Here underline indicated the restriction site, italic indicated His tag, bold indicated inker and bold underline indicated promoter sequence.

**Table S3.** Nucleic acid sequences for the construction of the mutants.

| DNA segments | Source |
| --- | --- |
| Neutral site for insertion  Upstream(slr0168)  AACTATGGCTTTGATGGTTATATGGGAATTCCCGGTATGGATGGCACCGATGCGGAATCCCAACAGATTGCCTTTGACAACAATGTGGCCTGGAATAACCTGGGGGATTTGTCCACCACCACCCAACGGGCCTACACTTCGGCTATTAGCACAGACACAGTGCAGAGTGTTTATGGCGTTAATCTGGAAAAAAACGATAACATTCCCATTGTTTTTGCGTGGCCCATTTTTCCCACCACCCTTAATCCCACAGATTTTCAGGTAATGCTTAACACGGGGGAAATTGTCACCCCGGTGATCGCCTCTTTGATTCCCAACAGTGAATACAACGAACGGCAAACGGTAGTAATTACGGGCAATTTTGGTAATCGTTTAACCCCAGGCACGGAGGGAGCGATTTATCCCGTTTCCGTAGGCACAGTGTTGGACAGTACTCCTTTGGAAATGGTGGGACCCAACGGCCCGGTCAGTGCGGTGGGTATTACCATTGATAGTCTCAACCCCTACGTGGCCGGCAATGGTCCCAAAATTGTCGCCGCTAAGTTAGACCGCTTCAGTGACCTGGGGGAAGGGGCTCCCCTCTGGTTAGCCACCAATCAAAATAACAGTGGCGGGGATTTATATGGAGACCAAGCCCAATTTCGTTTGCGAATTTACACCAGCGCCGGTTTTTCCCCCGATGGCATTGCCAGTTTACTACCCACAGAATTTGAACGGTATTTTCAACTCCAAGCGGAAGATATTACGGGACGGACAGTTATCCTAACCCAAACTGGTGTTGATTATGAAATTCCCGGCTTTGGTCTGGTGCAGGTGTTGGGGCTGGCGGATTTGGCCGGGGTTCAGGACAGCTATGACCTGACTTACATCGAAGATCATGACAACTATTACGACATTATCCT  Downstream(slr0168)  AAGCCGCAGTTCGCCAAATTAAGAGGGTTGCTTTGCCCTCCGAAGGGGATTATTCGGCGGTTTATAATCCCGGTGGCCCCGGCAATGATCCAGAGAATGGTCCCCCAGGGCCCTTTACTGTGTCCAGTAGTCCCCAGGTAATTAAGGTAACGGATACCATCGGCCAGCCCACCAAAGTCTCCTATGTGGAAGTGGATGGCCCCGTATTGCGTAATCCCTTCAGTGGTACTCCCATTGGGCAAGAGGTGGGTTTAGCGGTTAAAGATCTGGCCACAGGTCATGAAATTTATCAGTACACTGACCCAGATGGGAAGGTATTTTATGCTTCCTTTGCTGCCGCTGATGACCAAGCCACGGATTTAACCACGGCGATCGCCAATCCCACGGCCATCGATTTAATTAACGCCAGGGGATTTACGGCGGGTAGTTCCGTCACCGTATCGGGTTCCTACAGTCGGGAAGCCTTTTTTGATGGATCCATGGGTTTTTATCGACTTCTGGACGATAACGGTGCAGTGCTAGATCCCTTAACAGGTGGTGTAATCAACCCAGGACAGGTAGGTTATCAAGAAGCAGCTTTGGCAGATAGCAATCGTTTGCAAGCCACTGGCTCCACCCTAACGGCAGAAGACCTAGAAACCAGAGCATTTTCCTTCAATATTTTGGGTGGCGAGTTGTATGCGCCATTTTTAACGGTTAATGACAGTCTTTCCGGTATTAATCAGACTTATTTTGCCTTTGGGTCGGCCAACCCAGATGGCATCAGCCACAGCACAAACTTGGGACCCAACGTGATTGGTTTTGAAGATTTTCTCGGCGGAGGAG  PcpcB Promoter  GTTATAAAATAAACTTAACAAATCTATACCCACCTGTAGAGAAGAGTCCCTGAATATCAAAATGGTGGGATAAAAAGCTCAAAAAGGAAAGTAGGCTGTGGTTCCCTAGGCAACAGTCTTCCCTACCCCACTGGAAACTAAAAAAACGAGAAAAGTTCGCACCGAACATCAATTGCATAATTTTAGCCCTAAAACATAAGCTGAACGAAACTGGTTGTCTTCCCTTCCCAATCCAGGACAATCTGAGAATCCCCTGCAACATTACTTAACAAAAAAGCAGGAATAAAATTAACAAGATGTAACAGACATAAGTCCCATCACCGTTGTATAAAGTTAACTGTGGGATTGCAAAAGCATTCAAGCCTAGGCGCTGAGCTGTTTGAGCATCCCGGTGGCCCTTGTCGCTGCCTCCGTGTTTCTCCCTGGATTTATTTAGGTAATATCTCTCATAAATCCCCGGGTAGTTAACGAAAGTTAATGGAGATCAGTAACAATAACTCTAGGGTCATTACTTTGGACTCCCTCAGTTTATCCGGGGGAATTGTGTTTAAGAAAATCCCAACTCATAAAGTCAAGTAGGAGATTAATT | *Synechocystis* sp. PCC6803 |
| Kanamycin resistant gene(Km)  ACGCTCAGTGGAACGAAAACTCACGTTAAGGGATTTTGGTCATGAACAATAAAACTGTCTGCTTACATAAACAGTAATACAAGGGGTGTTATGAGCCATATTCAACGGGAAACGTCTTGCTCTAGGCCGCGATTAAATTCCAACATGGATGCTGATTTATATGGGTATAAATGGGCTCGCGATAATGTCGGGCAATCAGGTGCGACAATCTATCGATTGTATGGGAAGCCCGATGCGCCAGAGTTGTTTCTGAAACATGGCAAAGGTAGCGTTGCCAATGATGTTACAGATGAGATGGTCAGACTAAACTGGCTGACGGAATTTATGCCTCTTCCGACCATCAAGCATTTTATCCGTACTCCTGATGATGCATGGTTACTCACCACTGCGATCCCCGGGAAAACAGCATTCCAGGTATTAGAAGAATATCCTGATTCAGGTGAAAATATTGTTGATGCGCTGGCAGTGTTCCTGCGCCGGTTGCATTCGATTCCTGTTTGTAATTGTCCTTTTAACAGCGATCGCGTATTTCGTCTCGCTCAGGCGCAATCACGAATGAATAACGGTTTGGTTGATGCGAGTGATTTTGATGACGAGCGTAATGGCTGGCCTGTTGAACAAGTCTGGAAAGAAATGCATAAACTTTTGCCATTCTCACCGGATTCAGTCGTCACTCATGGTGATTTCTCACTTGATAACCTTATTTTTGACGAGGGGAAATTAATAGGTTGTATTGATGTTGGACGAGTCGGAATCGCAGACCGATACCAGGATCTTGCCATCCTATGGAACTGCCTCGGTGAGTTTTCTCCTTCATTACAGAAACGGCTTTTTCAAAAATATGGTATTGATAATCCTGATATGAATAAATTGCAGTTTCATTTGATGCTCGATGAGTTTTTC | plasmid pET28a |
| ssSec  TTTAAAGGAGGTTAAGTGTTAAACAAATCTGTTCAGATCCTCTCTGGAGTTGTGCTTGCTGCTGCGGCCTTAGGTTTTACAACCCCCGCCCAGGCT |  |
| Lac(beta-lactamase)  ATGAGTATTCAACATTTCCGTGTCGCCCTTATTCCCTTTTTTGCGGCATTTTGCCTTCCTGTTTTTGCTCACCCAGAAACGCTGGTGAAAGTAAAAGATGCTGAAGATCAGTTGGGTGCACGAGTGGGTTACATCGAACTGGATCTCAACAGCGGTAAGATCCTTGAGAGTTTTCGCCCCGAAGAACGTTTTCCAATGATGAGCACTTTTAAAGTTCTGCTATGTGGCGCGGTATTATCCCGTGTTGACGCCGGGCAAGAGCAACTCGGTCGCCGCATACACTATTCTCAGAATGACTTGGTTGAGTACTCACCAGTCACAGAAAAGCATCTTACGGATGGCATGACAGTAAGAGAATTATGCAGTGCTGCCATAACCATGAGTGATAACACTGCGGCCAACTTACTTCTGACAACGATCGGAGGACCGAAGGAGCTAACCGCTTTTTTGCACAACATGGGGGATCATGTAACTCGCCTTGATCGTTGGGAACCGGAGCTGAATGAAGCCATACCAAACGACGAGCGTGACACCACGATGCCTGCAGCAATGGCAACAACGTTGCGCAAACTATTAACTGGCGAACTACTTACTCTAGCTTCCCGGCAACAATTAATAGACTGGATGGAGGCGGATAAAGTTGCAGGACCACTTCTGCGCTCGGCCCTTCCGGCTGGCTGGTTTATTGCTGATAAATCTGGAGCCGGTGAGCGTGGGTCTCGCGGTATCATTGCAGCACTGGGGCCAGATGGTAAGCCCTCCCGTATCGTAGTTATCTACACGACGGGGAGTCAGGCAACTATGGATGAACGAAATAGACAGATCGCTGAGATAGGTGCCTCACTGATTAAGCATTGG | *Klebsiella pneumoniae* plasmid pJHCMW1 |
| FL3 flexible linker  ACTAGAGCTGAGGCCGCCGCAAAAGAAGCAGCAGCTAAGGAAGCTGCGGCGAAG | Synthesized |
| lgt Membrane protein (Prolipoprotein diacylglyceryl transferase)  GCCGAAGGAAGATTAATGATTGAGCAAATATTTTTCGGACAATTTCAGTCCCCCGGGCCGGTGATGTTCCAGATAGGGGGTTTTGCCCTGCGTTGGTACGGATTTTTGATTGCCAGTGCTGTCATTATTGGTTTGAATCTCTGTCAATGGTTGGGGCAAAAACGGGGCATTAACCCGGATTTATTCAACGATTTAGTCATTTGGTTAGTGGTGGCGGCCATCCCTTCTGCTCGCCTATATTACGTCGCCTTTGAGTGGCCCCGCTATGCCCAGCATTGGTTAAATATTTTTGCCATTTGGCAAGGGGGCATTGCTATCCATGGGGCCTTGATTGGGGGAACGATCGCCATTCTTGTTTTCAGTCGCTACCATCAGTTATCTTTCTGGAATTTGCTGGATGTACTCACCCCGGCGGTTATTCTCGGCCAGGCGATCGGTCGGTGGGGCAACTTTTTTAACTCCGAAGCTTTTGGTGCCCCCACTAATTTGCCTTGGAAGCTCTATATTCCCTTTGCTAATCGTCCGCTAAATCTGACCAGCTATGCCTATTTCCATCCTACTTTTTTATACGAATCAGTCTGGAACCTAGGAATTTTTGCAATCTTGATAGCCCTATTTTTTTATGGACTAAGAAATCCAGAAAAAATCAAAACTGGGACCATAACCTGTGTTTATTTGATTGGTTATAGCCTCGGTCGAGTGTGGATTGAAGGTTTAAGATTAGATAGTTTGATGCTTGGTCCTCTGAGAATAGCTCAGGTTGTTAGCATCACCCTAGTTTTATTGGGAACAGCGGGAATTGTCTGGTTATATCTTCTGCAGAAAAATTTACCGGACTGGTCGGAGCGAAAATTGGTAAAAAAT | *Synechocystis* sp. PCC 6803 |
| AcTesA(Acyl-CoA thioesterase I)  ATGAAAACCATTCTTATCTTAGGCGACAGTCTGAGTGCGGGTTATGGCATTAACCCCGAACAGGGCTGGGTCGCTTTATTACAAAAACGTCTGGATCAACAATTTCCCAAGCAGCATAAAGTCATTAATGCCAGTGTAAGTGGGGAAACCACCAGTGGTGCTTTAGCTCGTTTACCCAAACTACTTACTACTTATCGACCTAATGTGGTGGTCATTGAGCTTGGTGGTAATGATGCATTAAGAGGACAACCGCCTCAAATGATTCAAAGTAATCTGGAAAAATTAATCCAGCACAGCCAAAAGGCAAAATCTAAAGTCGTGGTGTTTGGAATGAAAATACCACCAAATTATGGCACTGCCTATAGTCAGGCATTTGAAAATAATTATAAGGTAGTGAGTCAAACATATCAGGTTAAGTTGTTGCCATTTTTTCTTGATGGTGTGGCTGGACACAAAAGTCTAATGCAAAATGACCAGATCCATCCAAATGCCAAAGCCCAGTCAATCTTGCTAAATAACGCATACCCATATATTAAAGGCGCTTTATAA | *Acinetobacter baylyi* |
| Upstream(slr1311)  ACCTTACTTGGATGCCGGTGCGATGTTGATAATCCCGAATCGCTTCCAATAGTCGTAGCGTCCCCATGGCCACTGAATCTACAGTGTATTCCGGAGAATCAAAGCTCACCCGCACGTGGGATTGGGCCCCCAGATTGTAAATCTCCGTCGGTTTGACATCTTCTAAAATGCGGCGCAGGGTGGTGCCGTCGGTCAGATCACCATAATGAAGTCGGAGTTTCGCCTCAAGATCATGGGGATCAACATAAAGATGATCAATGCGGTCAGTGTTAAAGGTAGAAGTTCGGCGAATGATGCCATGGACTTGGTAGCCCTTTTCCAACAACAATTCACTCAGATAGGAGCCATCTTGCCCCGTGATGCCTGTCAGCAAAACAACTTTAGACTTTGACATTAGTTAATTTTTCCCCATTGCCCCAAAATACATCCCCCTAAAAATATCAGAATCCTTGCCCAGATGCAGGCCTTCTGGCGATCGCCATGGTGAGCAACGATTGCGGCTTTAGCGTTCCAGTGGATATTTGCTGGGGGTTAATGAAACATTGTGGCGGAACCCAGGGACAATGTGACCAAAAAATTCAGGGATATCAATAAGTATTAG  Downstream(slr1311)  GGATAGCCAAGGCCGGGTAATCGGCACCTGGGCTGATGTATTGAACCGAGCCAACATCGGTTTTGAAGTAATGCACGAACGCAATGCCCACAACTTCCCCCTCGACTTAGCGTCTGGGGAGCAAGCTCCTGTGGCTTTGACCGCTCCTGCTGTCAACGGTTAATTCCTTGGTGTAATGCCAACTGAATAATCTGCAAATTGCACTCTCCTTCAATGGGGGGTGCTTTTTGCTTGACTGAGTAATCTTCTGATTGCTGATCTTGATTGCCATCGATCGCCGGGGAGTCCGGGGCAGTTACCATTAGAGAGTCTAGAGAATTAATCCATCTTCGATAGAGGAATTATGGGGGAAGAACCTGTGCCGGCGGATAAAGCATTAGGCAAGAAATTCAAGAAAAAAAATGCCTCCTGGAGCATTGAAGAAAGCGAAGCTCTGTACCGGGTTGAGGCCTGGGGGGCACCTTATTTTGCCATTAATGCCGCTGGTAACATAACCGTCTCTCCCAACGGCGATCGGGGCGGTTCGTTAGATTTGTTGGAACTGGTGGAAGCCCTGCGGCAAAGAAAGCTCGGCTTACCCCTATTAATTCGTTTTTCCGA | *Synechocystis* sp. PCC 6803 |
| Ptrc Promoter  GAGCTGTTGACAATTAATCATCCGGCTCGTATAATTTTAAAGAGGAGAAA | synthesized |

**Table S4**. List of genetically modified Cyanobacteria for biodiesel production.

| **Host** | **Genetic modification** | Production | **References** |
| --- | --- | --- | --- |
| Synechocystis sp. PCC6803 | Deletion of the slr1609 gene(acyl-ACP synthetase (Aas) | 6.7 mg/L /OD | ([Gao et al., 2012](#_ENREF_1)) |
| Synechocystis sp. PCC6803 | Aas deletion | 1.79 mg/L /day | ([Kaczmarzyk and Fulda, 2010](#_ENREF_5); [Quintana et al., 2011](#_ENREF_10)) |
| Synechoccocus PCC 7942 | Aas deletion | 2.35 mg/L /day | ([Kaczmarzyk and Fulda, 2010](#_ENREF_5); [Quintana et al., 2011](#_ENREF_10)) |
| Synechococcus sp. PCC 7002 | Introducing ‘tesA and inactivating aas | 40 mg/L | ([Ruffing, 2014](#_ENREF_11)) |
| Synechococcus elongatus PCC 7942 | Inactivating aas, overexpressed ‘tesA, deleting the nrtABCD genes and overexpress an RND-type efflux system | 45 mg/L | ([Kato et al., 2015](#_ENREF_7)) |
| Synechococcus elongatus PCC7942 | Introducing ‘tesA and inactivating aas | 49.3 mg/L | ([Ruffing and Jones, 2012](#_ENREF_12)) |
| Synechococcus elongatus PCC 7942 | Inactivating aas, overexpressed ‘tesA, deleting the nrtABCD genes, overexpress an RND-type efflux system and inactivating the wzt gene | 100 mg/L | ([Kato et al., 2016](#_ENREF_8)) |
| Synechococcus sp. PCC 7002 | Introducing ‘tesA and inactivating aas and overexpression of Rubisco | 131 mg/L | ([Ruffing, 2014](#_ENREF_11)) |
| Synechocystis sp. PCC6803 | Aas deletion, phosphotransacetylase gene deletion and codon-optimized tesA137 gene overexpression | 197 mg/L | ([Liu et al., 2011](#_ENREF_9)) |
| Synechocystis sp. PCC6803 | Acinetobacter baylyi thioesterase introduce on the inner membrane | 171 mg/L | This study |
| Synechococcus elongatus PCC 7942 | ([Kato et al., 2015](#_ENREF_7)) + two-phase culture system | 640 mg/L | ([Kato et al., 2017](#_ENREF_6)) |

**Supplementary methods**

**Method S1: Measurement of** **Chlorophyll a Concentration**

We measured Chl a content of WT, AcT and mAcT strain to understand their photosynthetic status. The cells were grown in corresponding growth conditions and the Chlorophyll a (Chl a) was isolated and measured following the method described by Sinetova *et.* al ([Sinetova et al., 2012](#_ENREF_13)). Briefly, optical density (OD) of cell culture measured at 730 and recorded. 1mL cells harvested from the WT, AcT and mAcT culture by centrifugation at 15,000 x g at room temperature for 7 min. The supernatant discarded and the cell pellet dissolved with precooled methanol. The sample then homogenized by vortexing or by gentle pipetting up and down. Then the samples were covered with aluminium foil and incubated for 20 min at 4°C. The supernatant was collected by centrifugation at 15,000 x g at 4 °C for 7 min. The Spectrophotometer calibrated using methanol as blank. Absorbance measured at OD_665_ and OD_720_. Three biological and three technical replications were considered for each strain. The concentration of chlorophyll a content calculated according to the following equation:

Chla [µg/ml] = 12.9447 (A665- A720)

The student’s t-test was performed to compare the Chl a content of WT, AcT and mAcT with each other and the p-value less than 0.05 considered as significant

**Method S2: Cellular ATP synthesis activity test**

ATP is an important element in a luciferase assay. The ATP concentration can be measured using this assay. Previously, Johnston et al. and hara and Mori ([Johnston et al., 2003](#_ENREF_4); [Hara and Mori, 2006](#_ENREF_3)) described a method of measuring intracellular ATP concentration by this assay. They used osmotic shock and detergent treatment to leak out the intracellular ATP into solution and then measured these ATPs by luciferase assay. To check the cellular ATP synthesis, 10 mL culture was harvested by centrifugation and the pellet washed by 100 mM Tris-HCl buffer (pH 7.4) and then suspended into the same buffer. The cell suspension mixed with an equal volume of pretreatment solution (40% [w/v] glucose, 0.8% concentrations of Triton X-100) and incubated 20 min. at room temperature (mixture A). Assay solution (0.5 mM D-luciferin, 1.25µg/ml firefly luciferase, 5 mM MgSO4, 100 mM EDTA, 1 mM dithiothreitol(DTT), and 25 mM Tricine buffer, pH 7.8) was prepared and incubated 15 mins at room temperature. A 1/20 volume of detergent solution (8%Triton X-100, 300 mM potassium phosphate, pH7.2) was added to the assay solution (mixture B). A 10µl “mixture A” added into 90µl of “mixture B” and then luminescence recorded at 0, 2, 4, 6 and 8 minutes. 10µl water added into 90µl of “mixture B” and considered as negative control.

10 ml of the supernatant that obtained from previous centrifugation kept on the ice was ﬁltered through a 0.45µm ﬁlter unit into a new tube. These cell-free solutions were used for the assay of leaked ATP due to membrane damaged in normal physiological condition. The 150µl supernatant mixed with 75µl of buffer (1.02%KH2PO4 +0.143% MgCl_2_ +0.0095% phosphoenolpyruvate, all adjusted to pH 7.3 and ﬁlter sterilized). Then this sample added with “mixture B” and luminescence was measured as mentioned above.

A standard curve was prepared using Luminescence at Y-axis and ATP concentration at X-axis. The luminescence recorded from the cell suspension and media sample was compared with the standard curve and the concentration of ATP calculated from the slope of linearly increasing luminescence.

**Method S3: Membrane Proteins isolation by Ultracentrifugation**

Membrane protein from WT, AcT and mAcT was isolated by following the method described by Haigh *et al.* ([Haigh et al., 2013](#_ENREF_2)). Cells harvested after induction of AcT at 24 h when the OD_730_ reached 0.45 by centrifugation for 10 min at 6700 rpm and 4 °C. The collected cell pellet washed twice in 100 mM Tris-HCl (pH 7.5) to a volume 50 times higher than that of the pellet, and the culture suspension was frozen at −80 °C for 2 h or overnight to weaken the cell wall. Cells were lysed by sonication in cycles of 15 s sonication, followed by 45 s cooling until the lysate changed from an opaque to a less turbid solution. Cell debris was removed from protein lysates by centrifugation for 10 min at 10000 × g at 4 °C. The resulting supernatant containing total cellular proteins was centrifuged at 100000 × g for 10 min at 4 °C to separate membrane proteins from cytosolic proteins using a TLA100 rotor in a Beckman Coulter L-100XP ultracentrifuge. The supernatant contained the cytoplasmic proteins, whereas the pellet contained the total membrane proteins. Protein concentration was measured using a Nanodrop spectrophotometer (Nanodrop 1000, Thermo Scientific) set at 280 nm. Proteins were separated by SDS-PAGE using 10% polyacrylamide gel. An equal amount of protein was loaded into the wells of the gel. A protein marker is also loaded. The gel after running blotted onto PVDF membranes to detect the target protein with the specific anti-His antibody.

**Supplementary references**

Gao, Q., Wang, W., Zhao, H., and Lu, X. (2012). Effects of fatty acid activation on the photosynthetic production of fatty acid-based biofuels in Synechocystis sp. PCC6803. *Biotechnol Biofuels* 5(1)**,** 17. doi: 10.1186/1754-6834-5-17.

Haigh, R., Kumar, B., Sandrini, S., and Freestone, P. (2013). Mutation design and strain background influence the phenotype of Escherichia coli luxS mutants. *Mol Microbiol* 88(5)**,** 951-969. doi: 10.1111/mmi.12237.

Hara, K.Y., and Mori, H. (2006). An efficient method for quantitative determination of cellular ATP synthetic activity. *J Biomol Screen* 11(3)**,** 310-317. doi: 10.1177/1087057105285112.

Johnston, M.D., Hanlon, G.W., Denyer, S.P., and Lambert, R.J. (2003). Membrane damage to bacteria caused by single and combined biocides. *J Appl Microbiol* 94(6)**,** 1015-1023.

Kaczmarzyk, D., and Fulda, M. (2010). Fatty acid activation in cyanobacteria mediated by acyl-acyl carrier protein synthetase enables fatty acid recycling. *Plant Physiol* 152(3)**,** 1598-1610. doi: 10.1104/pp.109.148007.

Kato, A., Takatani, N., Ikeda, K., Maeda, S.I., and Omata, T. (2017). Removal of the product from the culture medium strongly enhances free fatty acid production by genetically engineered Synechococcus elongatus. *Biotechnol Biofuels* 10**,** 141. doi: 10.1186/s13068-017-0831-z.

Kato, A., Takatani, N., Use, K., Uesaka, K., Ikeda, K., Chang, Y., et al. (2015). Identification of a Cyanobacterial RND-Type Efflux System Involved in Export of Free Fatty Acids. *Plant Cell Physiol* 56(12)**,** 2467-2477. doi: 10.1093/pcp/pcv150.

Kato, A., Use, K., Takatani, N., Ikeda, K., Matsuura, M., Kojima, K., et al. (2016). Modulation of the balance of fatty acid production and secretion is crucial for enhancement of growth and productivity of the engineered mutant of the cyanobacterium Synechococcus elongatus. *Biotechnol Biofuels* 9**,** 91. doi: 10.1186/s13068-016-0506-1.

Liu, X., Sheng, J., and Curtiss, R., 3rd (2011). Fatty acid production in genetically modified cyanobacteria. *Proc Natl Acad Sci U S A* 108(17)**,** 6899-6904. doi: 10.1073/pnas.1103014108.

Quintana, N., Van der Kooy, F., Van de Rhee, M.D., Voshol, G.P., and Verpoorte, R. (2011). Renewable energy from Cyanobacteria: energy production optimization by metabolic pathway engineering. *Appl Microbiol Biotechnol* 91(3)**,** 471-490. doi: 10.1007/s00253-011-3394-0.

Ruffing, A.M. (2014). Improved Free Fatty Acid Production in Cyanobacteria with Synechococcus sp. PCC 7002 as Host. *Front Bioeng Biotechnol* 2**,** 17. doi: 10.3389/fbioe.2014.00017.

Ruffing, A.M., and Jones, H.D. (2012). Physiological effects of free fatty acid production in genetically engineered Synechococcus elongatus PCC 7942. *Biotechnol Bioeng* 109(9)**,** 2190-2199. doi: 10.1002/bit.24509.

Sinetova, M.A., Cerveny, J., Zavrel, T., and Nedbal, L. (2012). On the dynamics and constraints of batch culture growth of the cyanobacterium Cyanothece sp. ATCC 51142. *J Biotechnol* 162(1)**,** 148-155. doi: 10.1016/j.jbiotec.2012.04.009.
